# Material-engineered bioartificial microorganisms enabling efficient scavenging of waterborne viruses

**DOI:** 10.1038/s41467-023-40397-5

**Published:** 2023-08-03

**Authors:** Huixin Li, Yanpeng Xu, Yang Wang, Yihao Cui, Jiake Lin, Yuemin Zhou, Shuling Tang, Ying Zhang, Haibin Hao, Zihao Nie, Xiaoyu Wang, Ruikang Tang

**Affiliations:** 1 Department of Chemistry, Zhejiang University, Hangzhou, Zhejiang China; 2 Department of Cardiology, Sir Run Run Shaw Hospital, School of Medicine, Zhejiang University, Hangzhou, Zhejiang China; 3 Qiushi Academy for Advanced Studies, Zhejiang University, Hangzhou, Zhejiang China; 4 Key Laboratory of Cardiovascular Intervention and Regenerative Medicine of Zhejiang Province, Hangzhou, Zhejiang China; 5 Laboratory of Virology, Beijing Key Laboratory of Etiology of Viral Diseases in Children, Capital Institute of Pediatrics, Beijing, China; 6 School of Chemistry and Chemical Engineering, Nantong University, Nantong, Jiangsu China

**Keywords:** Bioinspired materials, Water microbiology, Biocatalysis

## Abstract

Material-based tactics have attracted extensive attention in driving the functional evolution of organisms. In aiming to design steerable bioartificial organisms to scavenge pathogenic waterborne viruses, we engineer *Paramecium caudatum* (Para), single-celled microorganisms, with a semiartificial and specific virus-scavenging organelle (VSO). Fe_3_O_4_ magnetic nanoparticles modified with a virus-capture antibody (MNPs@Ab) are integrated into the vacuoles of Para during feeding to produce VSOs, which persist inside Para without impairing their swimming ability. Compared with natural Para, which has no capture specificity and shows inefficient inactivation, the VSO-engineered Para (E-Para) specifically gathers waterborne viruses and confines them inside the VSOs, where the captured viruses are completely deactivated because the peroxidase-like nano-Fe_3_O_4_ produces virus-killing hydroxyl radicals (•OH) within acidic environment of VSO. After treatment, magnetized E-Para is readily recycled and reused, avoiding further contamination. Materials-based artificial organelles convert natural Para into a living virus scavenger, facilitating waterborne virus clearance without extra energy consumption.

## Abstract (web summary)

The material-based evolution of organisms has attracted broad interdisciplinary interest, however, the fabrication of material-integrated organelles remains inadequately exploited. Here the authors engineer a bioartificial organism by integrating a semiartificial and specific virus-scavenging organelle to scavenge pathogenic waterborne viruses.

## Introduction

The integration of functional nanomaterials and organisms can promote the functional evolution of living organisms with addressable biological responsiveness and broad application prospects^1–3^, and it thus attracts extensive attention in biomedicine^4–6^, microrobot fabrication^7–9^, energy conversion^10,11^ and environmental science^12^. Intriguingly, magnetotactic bacteria (MTB) are typical examples of organisms that regulate their own biological functions using magnetic materials^13,14^. MTB feature organelles known as magnetosomes that contain magnetic nanoparticles enveloped by lipid bilayers, which play a vital role in the maintenance of magnetotaxis and survival of the MTB^15,16^. Of note, the compartment for the magnetosome acts as a potential gate for differentiation of the pH or redox potential between the vesicle and the cellular environment^17^. Inspired by MTB, dynamic subcellular compartments are favorable for material integration since they can shield biological clearance while maintaining relative stability in the intracellular environment, representing a key element for organism modification. However, although the material-based evolution of organisms has attracted broad interdisciplinary interest, the strategies needed to fabricate the abovementioned material-integrated organelles remain inadequately exploited.

Protists are the most important grazers of viruses in aquatic environments. They play essential roles in the effective control of biological waste-water^18,19^. Attempts have been made to use ciliates to address water environment problems^20^. However, susceptibility of the virus to grazing by protists is highly dependent on the species and hydrophobicity of the virus^21^. Despite the promise shown for using protists to treat waterborne viruses, their removal rates are restricted by less than 4 orders of magnitude because of the lack of mechanistic insight. More importantly, due to low efficiency in inactivating viruses, protists can also act as a virus reservoir to shield the ingested viruses from inactivation treatment^22^, which increases the transmission risk of waterborne virus. According to data from World Health Organization, waterborne disease caused by viruses still outbreaks worldwide despite decades of development of membrane filtration and disinfection technologies^23^, such as reverse osmosis (RO), ultra-filtration (UF), nanofiltration (NF,) and chlorination/UV/ozone (Supplementary Table 1)^24–27^. In contrast to conventional techniques, we propose the design of a bioartificial virus-scavenging organelle (VSO) to endow protists with the ability to specifically capture and eliminate waterborne virus.

**Fig. 1:**
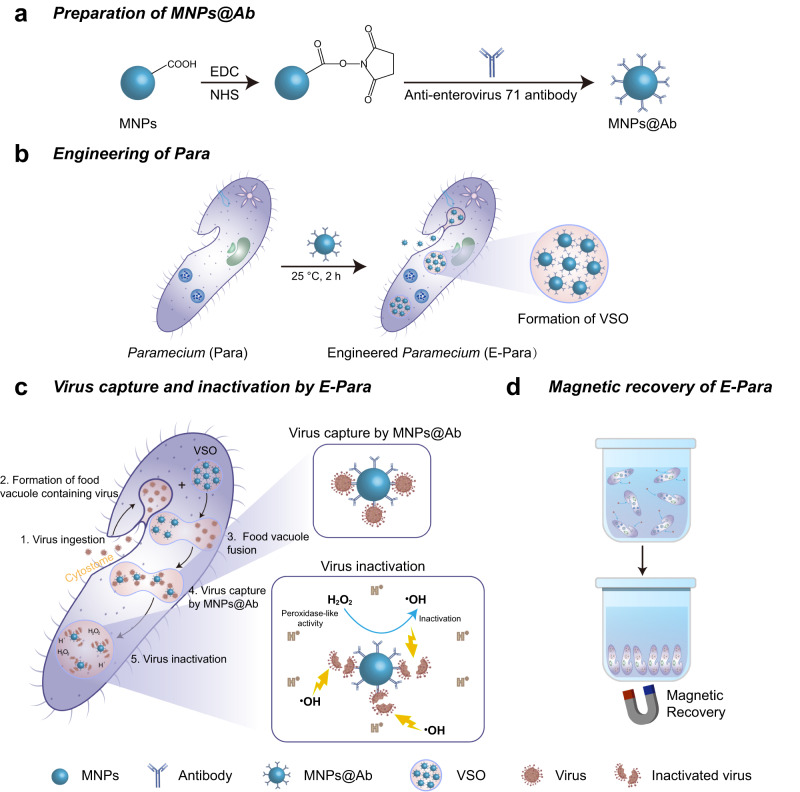
Schematic illustration of the preparation and working principle of E-Para. **a** Preparation of MNPs@Ab. **b** The formation of VSO after engineering of Para using MNPs@Ab. **c** The proposed mechanism of virus capture and inactivation by E-Para. The virus capture by E-Para involves four processes: 1. Virus ingestion; 2. Formation of food vacuole containing virus; 3. Food vacuole fusion; 4. Virus capture by MNPs@Ab. The virus inactivation by E-Para was shown as 5. Virus inactivation. **d** Magnetic recovery of E-Para.

We use *Paramecium caudatum* (Para), a single-celled free-living ciliate, as a model, because Para can reproduce, swim in viscous water, and graze and consume chloroviruses as food while avoiding the production of toxic byproducts and high energy costs^28,29^. However, although a few studies reported that Para are able to ingest certain kind of viruses, the virus-capture and virus-elimination ability is virus-specific and less effective^22,28,29^. Thus, it is impossible to directly use either Para or protists as a strategy to remove viruses. Herein, we take advantage of the food ingesting process of Para^30^ to enable the construction of artificial subcellular organelles inside the Para^31,32^. Accordingly, we design a Fe_3_O_4_ magnetic nanoparticle modified with a virus-targeted antibody (MNPs@Ab, Fig. 1a) and integrate MNPs@Ab into vacuoles via a feed process to introduce a specific virus targeting and scavenging organelle (VSO, Fig. 1b). The obtained VSO modules had a long-life span inside the engineered Para (E-Para) and enabled virus capture by the presence of specific antibodies in VSOs via the fusion of virus-loaded vacuoles and VSOs (Fig. 1c). Inside VSOs, the acidic environment containing a large amount of hydrogen peroxide (H_2_O_2_) stimulated the peroxidase-like activity of MNPs^33^ and generated hydroxyl radicals via the Fenton reaction^34^, leading to efficient deactivation of viruses. After capturing the viruses, the E-Para were efficiently collected with an external magnet to minimize environmental pollution (Fig. 1d). To achieve a versatile VSO for removal of generic virus, antibodies on the MNPs@Ab were replaced by virus capturing sialic acid, which simultaneously grazes three different kinds of viruses in water, reflecting the universality of this strategy. The E-Para scavenges pathogenic viruses from environmental water, representing a promising biorobot to control waterborne diseases and purify environmental water. Our findings provide a new concept for promoting the functional evolution of living organisms with semiartificial organelles engineered by functional nanomaterials.

## Results

### Engineering of Para by VSO

Fe_3_O_4_ magnetic nanoparticles (MNPs) were synthesized by a solvothermal method using sodium citrate as a modifier. The powder X-ray diffraction (pXRD) spectra confirmed the crystallinity of the obtained MNPs (Supplementary Fig. 1). Since the surface chemistry of MNPs may influence their stability in Para and their cytotoxicity toward Para, we employed sodium citrate and polymers, including polyethyleneimine (PEI), polyethylene glycol (PEG), and polyacrylic acid (PAA), to modify the MNPs, which were then incubated with Para for 2 h. As indicated by the remaining Fe content inside the Para (Supplementary Fig. 2) and the cytotoxicity assay (Supplementary Fig. 3), sodium citrate-modified Fe_3_O_4_ (Fe_3_O_4_@sodium citrate) enabled efficient in vivo retention and showed less cytotoxicity than the other modifiers. Thus, trisodium citrate dihydrate was added during the synthesis of the MNPs before antibody modification. The successful coating of sodium citrate on the MNP surface was confirmed by examining the characteristic peaks of the C–O stretching vibrations at 1396 cm^−1^, C = O stretching vibrations at 1597 cm^−1^ and O–H stretching vibrations at 3416 cm^−1^ after modification using Fourier transform infrared (FTIR) spectroscopy (Supplementary Fig. 4). The carboxy group from sodium citrate serves as a reactive site for antibody modification. The EV71 monoclonal antibody was attached onto the surface of MNPs using *N*-ethyl-*N*′-(3-dimethylaminopropyl) carbodiimide (EDC)/*N*-hydroxysuccinimide (NHS) conjugation chemistry. Upon antibody conjugation, the zeta potential of MNPs@Ab shifted from −6 to −12 mV (Supplementary Fig. 5).

**Fig. 2:**
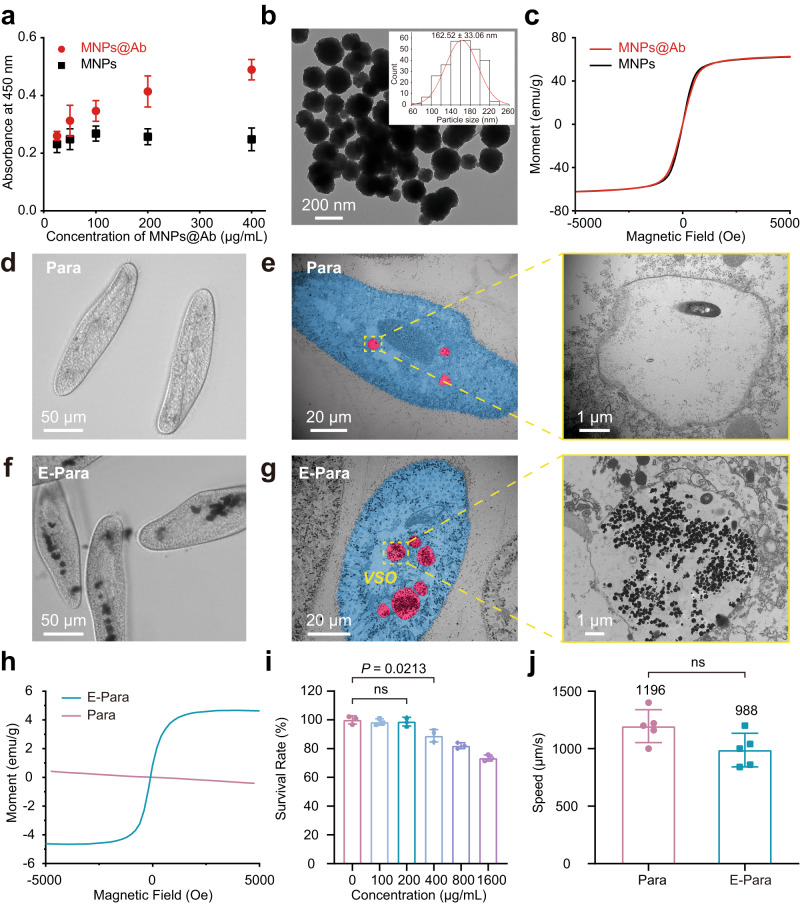
Construction and characterization of E-Para. **a** ELISA of MNPs@Ab confirmed the antibody conjugation. The data are presented as the mean ± sd (*n*  =  3). **b** TEM image of MNPs@Ab and size distribution of MNPs@Ab (inset). **c** Magnetic hysteresis loops of MNPs@Ab and MNPs. **d**, **e** Optical microscopy **d** and TEM images **e** of the food vacuoles in natural Para. The highlighted vacuole area was enlarged. **f**, **g** Optical microscopy **f** and TEM images **g** of the food vacuoles of E-Para. The highlighted VSO area was enlarged. **h** Magnetic hysteresis loops for natural Para and E-Para. **i** For fabrication of E-Para, Para were cocultured with different concentrations of MNPs@Ab for 2 h. The obtained E-Para were then cultured in medium without feeding for another 24 h for toxicity evaluation. The data are presented as the mean ± sd (*n* = 3). **j** Speeds of natural Para and E-Para. The data are presented as the mean ± sd (*n* = 3). In **i**, **j**, statistical significance was calculated via two-tailed Student’s t test. *P*  <  0.05 was considered significant. Ns not significant.

The virus binding affinity of MNPs@Ab alone was first examined by using an enzyme-linked immunosorbent assay (ELISA) to confirm that MNPs@Ab could recognize viruses. The elevated OD seen upon adding MNPs@Ab suggested MNPs@Ab preserved its binding affinity toward EV71 (Fig. 2a). Transmission electron microscopy (TEM) analysis showed dimensions of MNPs@Ab with a diameter of ∼162 nm (Fig. 2b). Moreover, the MNPs@Ab showed a saturation magnetization of ∼ 62 emu/g, which was comparable to that of the MNPs control in the presence of antibody (Fig. 2c).

The in vivo VSO was engineered by feeding Para with MNPs@Ab (200 μg/mL)-containing modified Dryl’s solution (named KDS buffer, a phosphate buffer commonly used in *Paramecium* studies)^35^ for 2 hours at 25 °C. First, we used phase contrast microscopy to observe the E-Para before and after incubation with MNPs@Ab. As shown, natural Para showed transparent vacuoles while E-Para displayed dark and isolated vacuole-like structures inside the cells (Fig. 2d, f), which indicated the entrance of MNPs@Ab during the feeding process. TEM was then used to verify the subcellular distribution of MNPs@Ab. The vacuoles of natural Para were almost empty due to starvation (Fig. 2e), whereas all of the vacuoles of E-Para were filled with large amounts of MNPs@Ab (Fig. 2g). No significant difference was observed between the size of the as-prepared MNPs@Ab and ingested MNPs@Ab in E-Para (in vivo) (Supplementary Fig. 6), indicating the stability of MNPs@Ab. Furthermore, the magnetic hysteresis loop for E-Para showed superparamagnetic characteristics similar to those of MNPs@Ab, while natural Para was diamagnetic (Fig. 2h). The intracellular MNPs@Ab were then quantitatively evaluated using inductively coupled plasma‒mass spectrometry (ICP‒MS), which showed 30.06 ± 2.44 μg Fe per 10^4^ E-Para cells (Supplementary Fig. 7). These results confirmed the effective in vivo formation of VSOs inside Para (E-Para). Furthermore, the MNPs@Ab-laden vacuoles were stable in Para for at least 24 h, exhibiting the stability of VSO in E-Para (Supplementary Fig. 8).

The effect of VSO on the biological properties of E-Para was further examined. To assess the cytotoxicity of the implanted VSOs, we calculated the survival rate^36^ of Para after coincubation with a series concentration of MNPs@Ab. The results showed that MNPs@Ab exhibited minimized cytotoxicity to Para at concentrations up to 200 μg/mL, manifesting acceptable biocompatibility (Fig. 2i). To estimate if the MNPs@Ab affect the athletic performance of Para, we evaluated the movement speed of the E-Para. Compared with that of natural Para (Supplementary Movie 1), the speed of the E-Para decreased slightly, which might be attributed to the increased weight of the Para due to the VSO implantation, but there was no significant difference according to the swimming speed (Fig. 2j) and direction (Supplementary Movie 2). These results indicated that the E-Para remained viable after engineering.

### Virus capture by E-Para

**Fig. 3:**
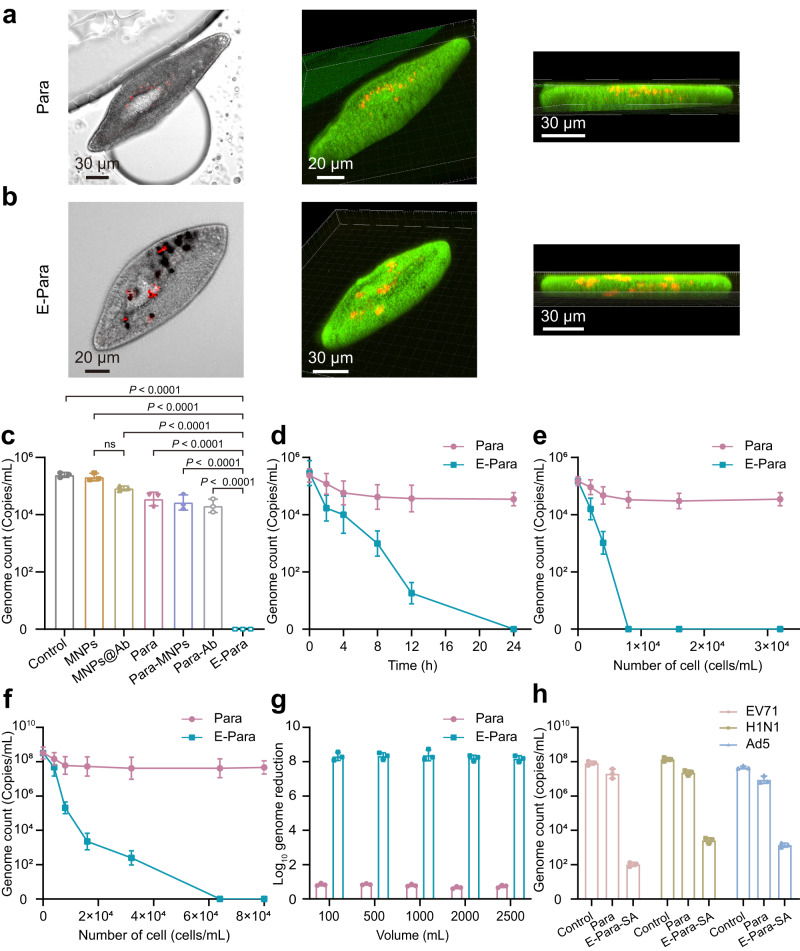
Virus capture by E-Para. **a**, **b** Phase and CLSM images of Para **a** and E-Para **b** after capturing the EV71. In vivo EV71 was localized by merging the phase and fluorescence images. Green represented CMFDA-labeled Para and red represented AF555-labeled EV71. **c** To confirm the virus capture capacity of MNPs, MNPs@Ab, Para, Para modified with MNPs (Para-MNPs), Para fed with antibody (Para-Ab) and E-Para, the remaining EV71 in the water was examined after coculturing the above samples with EV71 for 24 h. The data are presented as the mean ± sd (*n* = 3). Statistical significance was calculated via two-tailed Student’s t test. *P* < 0.05 was considered significant. Ns not significant. **d** Time-dependent virus capture by Para and E-para. The data are presented as the mean ± sd (*n* = 3). **e** Amount of viral genome remaining in EV71-contaminated water (1.5 × 10^5^ copies/mL) after treatment with different amounts of Para and E-Para for 24 h. The data are presented as the mean ± sd (*n* = 3). **f** Amount of viral genome remaining in EV71-contaminated water (3.2 × 10^8^ copies/mL) after treatment with different amounts of Para and E-Para for 24 hours. The data are presented as the mean ± sd (*n*  =  3). **g** The of log_10_ reductions in viral genome levels were calculated after different volumes EV71 solutions were treated with E-Para or Para (6.4 × 10^4^ cells/mL). **h** For generic virus removal, MNPs were modified by sialic acid (SA), which simultaneously grazed EV71, H1N1, and Ad5 from solution, reflecting the versatility of this strategy. The data are presented as the mean ± sd (*n* = 3).

Then ingestion of virus was investigated by placing E-Para or Para (8 × 10^3^ cells) in 1 mL of EV71 (10^5^ PFU/mL) for 4 hours at 25 °C. The Para and ingested viruses were then observed by using confocal laser scanning microscopy (CLSM). Although red dye-labeled EV71 was observed in both groups, E-Para captured more viruses than the natural Para, demonstrating that VSO enhanced the virus capture capacity (Fig. 3a, b). Moreover, we found that the red signals of the viruses were completely colocalized in the VSOs inside the E-Para (Fig. 3b). Three-dimensional construction images of the E-Para also convinced that the viruses were localized inside the cells but not absorbed on the cell surfaces. We also used cross-sectional views of E-Para to confirm that the EV71 was captured by MNPs@Ab (Supplementary Fig. 9).

To determine whether the viral capture was dependent on antibodies on the MNPs, we compared the virus capture efficiencies of MNPs, MNPs@Ab, Para engineered by MNPs without antibody modification (Para-MNPs), or Para fed with antibody alone (Para-Ab) with that of E-Para by determining the viral genome remaining in suspension after 24 h of incubation. The presence of antibody on MNPs@Ab showed no significant enhancement of virus capture since MNPs is instable and tend to aggregate in solution^37^, leading to reduced virus binding affinity (Fig. 3c). Besides, for Para-Ab, Para-MNPs, and native Para, their virus-capture ability remains at low level than that of E-para (Fig. 3c). For Para-Ab, the antibody was unable to reach a high local concentration in the vacuoles. Thus, the ingested viruses were not efficiently retained in vacuoles and could be excreted. In contrast, ingestion of MNPs@Ab in VSO resulted in a long-term retention and enrichment of antibody inside E-Para, which resulted in high virus capture efficiency. This result confirmed that the attachment of virus-specific antibody on MNPs significantly enabled the ability of VSO to capture viruses.

The virus-capturing efficiencies of E-para or Para over time were then explored. For Para, 8 to 24 h of capture led to only a 0.8 log_10_ reduction in the amount of viral genome. In contrast, the VSOs of E-Para drastically improved the capture efficiency over time and eventually led to a complete scavenge of viral genome after 24 h (Fig. 3d). The remaining viruses in the supernatant were also validated using indirect immunofluorescence analysis (IFA). These results showed that number of infected rhabdomyosarcoma (RD) cells was reduced after treatment with E-Para (Supplementary Fig. 10), which was consistent with the RT‒qPCR results, revealing that E-Para captured the virus more effectively than Para. These results showed that VSO plays important roles in the virus capture process. The current challenge for waterborne disease control is that the small sizes and very low concentrations in environmental water make virus clearance with conventional filter devices extremely difficult. However, we found that E-para showed high efficacy for capturing viruses with smaller sizes (approximately 30 nm) and with lower concentration in an effective and environmentally friendly manner without the need for extra devices.

The dosage-dependent virus scavenging behavior of E-Para showed that EV71 at a concentration of 1.5 × 10^5^ copies/mL was completely removed by 8 × 10^3^ cells/mL of E-Para within 24 h after incubation (Fig. 3e). The minimum concentration needed to clear these viruses was 8 × 10^3^ cells/mL of E-Para (Fig. 3e). Of note, when the virus concentration was increased to 3.2 × 10^8^ copies/mL, at least 6.4 × 10^4^ cells/mL E-Para were needed to completely clear these viruses, indicating dosage-dependent virus removal (Fig. 3f). In contrast, an increase in the level of native Para did not significantly improve virus removal (Fig. 3e, f), which was consistent with previous results^21^. The infectivity of the EV71-contaminated solution treated by E-Para was also measured with a plaque-forming assay (Supplementary Fig. 11). Furthermore, the feasibility of VSO-based virus capture was determined by increasing the volumes of the EV71-containing solutions. With increases in the water volume, the E-Para treatment resulted in an 8 log_10_ reduction in the viral genome, exceeding the WHO standard (4 log_10_ reduction value) (Fig. 3g)^38^. Moreover, the removal efficacy showed no obvious decrease when the volume was raised to 2500 mL, indicating preferable processing capacity in large volumes of water.

Antibody-based virus-specific capture is not generic to other types of waterborne viruses. In case of purifying water of the broad variety of waterborne viruses, equipping the E-Para with VSO that targets different types of potential viruses is also required. Therefore, we modified the MNPs with sialic acid (SA), a monosaccharide and a key component for receptor attachment to viruses such as enterovirus, influenza, and adenovirus^39–41^, to verify the versatility of the VSO-based strategy. After modification of SA, the zeta potential of the MNPs@SA shifted from 26.6 to −9.1 mV (Supplementary Fig. 12a). The SA-modified MNPs (MNPs@SA) exhibited the characteristic FTIR peaks for N–H stretching vibrations at 3385 cm^−1^, C = O stretching vibrations at 1617 cm^−1^, N–H bending vibrations at 1541 cm^−1^, C–O–C asymmetric stretching vibrations at 1278 cm^−1^ and C–O–C symmetric stretching vibrations at 1068 cm^−1^ (Supplementary Fig. 12b).

Para was engineered by MNPs@SA in the same way as E-Para and was named E-Para-SA. The E-Para-SA was used to treat a solution containing EV71 (8.2 × 10^7^ copies/mL), H1N1 (1.4 × 10^8^ copies/mL) and Ad5 (4.6 × 10^7^ copies/mL). Due to the attachment of the viruses to glycans of the sialic acid, the log_10_-reduction levels for EV71, H1N1 and Ad5 by E-Para-SA were 6, 5, and 5 respectively (Fig. 3h), indicating significantly higher virus-capturing efficiency than native Para. The removal efficiencies for the three viruses reached 99.99%, which were more than 4 log_10_ copies/mL. These data indicated generic virus-capturing ability, suggesting the versatility of VSO can be tailored by rational design of functional groups on the MNPs.

### Mechanism of virus capture by E-Para

How does MNPs@Ab-containing VSOs capture viruses? CLSM images of virus capture by E-Para showed the co-localization of viruses (red) and MNPs@Ab (black in phase image) in the same vacuoles of E-Para (Fig. 3b). This result verified the newly ingested viruses entered the pre-existing VSOs. To investigate how food pass from new vacuoles to VSO of E-Para, we incubated E-Para (Supplementary Fig. 13a, b) with *Escherichia coli* (*E.coli*), and observed the formation of vacuoles contained both bacteria and MNPs@Ab at the same time. *E.coli* were ingested by E-Para through cytostome (Supplementary Fig. 13c) to form a new food vacuole (FV) marked in blue (Supplementary Fig. 13d). We found *E.coli*-containing vacuoles fused with MNPs@Ab-containing VSO, indicating the food passage by a vacuole’s fusion pathway (Supplementary Fig. 13e–h). These results showed the virus capture by VSO is based on the food passage of Para, by which virus-containing vacuoles fused with circulating VSOs in vivo. After entering the VSO, the viruses were captured by antibodies in VSO (Fig. 3c). If antibody is not attached on MNPs, they were unable to be entrapped and concentrated inside vacuoles of Para for long time, resulting in low level of virus capture (Fig. 3c). In contrast, the long-term retention and enrichment of MNPs@Ab in VSO resulted in a high local antibody concentration in vacuoles (Supplementary Fig. 8), which is beneficial for virus capture and entrapment by antibodies inside VSO, thus improving the virus-capture efficiency of E-Para (Fig. 3c).

### Inactivation of viruses by E-Para

**Fig. 4:**
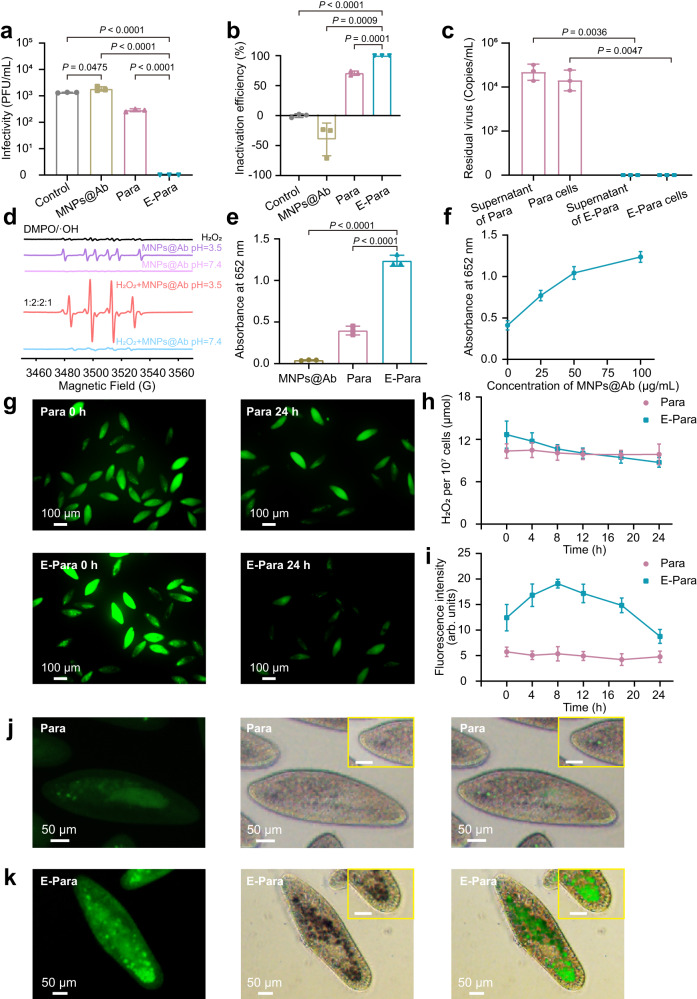
Virus inactivation effects of VSO. **a** Intra-Para EV71 titer detected with the plaque-forming assay. **b** Inactivation efficiencies of MNPs@Ab, Para and E-Para. **c** Viral genome remaining inside E-Para or Para cells after treatment. **d** EPR spectra of MNPs@Ab at different pH values. **e** Comparison of the catalytic capacities of MNPs@Ab, natural Para and E-Para to TMB. **f** The dose-dependent catalytic capacity of E-Para. **g** Fluorescence images of Para and E-Para stained with ROSGreen^TM^ (a H_2_O_2_ probe with green fluorescent). **h** H_2_O_2_ contents in Para and E-Para. **i** Fluorescence intensity of •OH measured by ImageJ. The data are presented as the mean ± sd (*n*  =  3). **j**, **k** Fluorescence images of Para **j** and E-Para **k** stained with HPF (a •OH probe with green fluorescent). The images in the yellow boxes are partially enlarged (Bar, 20 μm). Statistical comparisons were made using either two-tailed Student’s t test (**a**, **b**, **e**) or two-way analyses of variance (ANOVA) with Tukey’s multiple-comparison test **c**. *P* < 0.05 was considered significant. Ns not significant.

Virus inactivation by E-Para was assessed by examining the infectivity of E-Para-captured viruses using plaque-forming assays. After EV71 was captured for 24 h, the viruses inside Para and E-Para were released by cell lysis treatments and then used to infect RD cells to determine the remaining infectivity. As a control, cell lysis treatment with the SDS lysis buffer showed little effect on viral infectivity (Supplementary Fig. 14). Of note, the viruses ingested by E-Para completely lost their infectivity (Fig. 4a), which indicated that the E-Para not only captured the viruses but also inactivated them. In contrast, the titer of viruses captured by natural Para were reduced by less than 1 log_10_ PFU/mL, suggesting that the viruses were not completely inactivated by natural Para (Fig. 4a). Additionally, MNPs@Ab alone had no virucidal effect in water (Fig. 4a). The inactivation of ingested viruses by E-Para approached 100%, while natural Para and MNPs@Ab showed 70.87% and no inactivation respectively (Fig. 4b). Taken together, these data indicate that the viruses remained infectious inside the vacuoles of native Para or after treatment with MNPs@Ab, while the VSOs inside E-Para completely inactivated the infested viruses, implying a synergetic deactivation effect of MNPs@Ab in vacuoles. Para shielded the viruses and viral genomes inside the vacuoles, which remained infectious. We then investigated whether the E-Para or Para completely inactivated the ingested virus after incubation with EV71 for 24 h. For natural Para, the ingested virus remained infective and the viral genome was detectable, indicating the potential risk for using natural for virus capture. Nonetheless, for E-Para, no residual viral genome was detected in VSO (Fig. 4c). These results indicated that E-Para effectively scavenged the ingested viruses inside the VSOs.

### Hydroxyl radical-based virus deactivation mechanism

Since ferric oxide has peroxidase-like activity in acidic pH environments, the MNPs@Ab in the VSOs is capable of generating hydroxyl radical (•OH) by catalyzing hydrogen peroxide (H_2_O_2_) through Fenton-like reaction^34^. •OH can inactivate viruses because it reacts with almost all types of biomolecules, such as lipids and nucleotides^42,43^. To investigate the peroxidase-like activity of MNPs@Ab in vitro, electron paramagnetic resonance (EPR) spectroscopy was used. At pH 3.5, MNPs@Ab displayed stronger dose-dependent EPR signals (1:2:2:1) with DMPO/•OH (Supplementary Fig. 15) in the presence of H_2_O_2_ than it did without added H_2_O_2_ (Fig. 4d). Nevertheless, no •OH signal was detected at pH 7.4 for MNPs@Ab or the mixture of MNPs@Ab and H_2_O_2_ (Fig. 4d). Therefore, in acidic solutions containing H_2_O_2_, MNPs@Ab generated highly reactive •OH.

In order to verify the antiviral ability of MNPs@Ab-induced •OH in vitro, we incubated MNPs@Ab (200 μg/mL) with EV71 in the presence of H_2_O_2_ (1 mM) at different pH for 24 h and examined remained titer of EV71 by plaque assay. In acidic solutions containing H_2_O_2_, infectivity of EV71 was effectively reduced by MNPs@Ab (Supplementary Fig. 16), due to the generation of highly reactive •OH (Fig. 4d and Supplementary Fig. 15). On the contrary, under pH of 6, 7, and 8, the MNPs@Ab was unable to inactivate EV71.

We also tested the peroxidase-like activity of MNPs@Ab inside the VSOs in vivo. 3,3,5,5-Tetramethylbenzidine (TMB), a substrate of peroxidase, reacts with ferric oxide in the presence of H_2_O_2_ under acidic conditions to develop a blue color with a maximum absorbance at 652 nm^33^. We therefore added TMB to KDS buffer containing E-Para to look for the blue product. As expected, both Para and E-Para cells produced a blue catalysate in vivo (Supplementary Fig. 17), while the absorbance at 652 nm for TMB-treated E-para was evidently stronger than that of natural Para (Fig. 4e), indicating a reinforced catalytic reaction occurred inside E-Para. Notably, the enzyme activity increased as the concentration of MNPs@Ab increased, confirming that the catalytic effect was indeed related to MNPs@Ab in the VSOs (Fig. 4f).

To inspect the presence of H_2_O_2_ inside Para, ROSGreen^TM^, a special H_2_O_2_ probe was used that emits green fluorescence upon contact with H_2_O_2_. Both Para and E-Para exhibited the presence of intracellular H_2_O_2_ (Fig. 4g), which was mainly derived from lysosomes or peroxisomes in the cytoplasm^44^. Due to the presence of MNPs@Ab, the H_2_O_2_ levels in the VSOs elevated more rapidly than they did with Para within the first 8 h, and then decreased faster than they did with Para, suggesting accelerated H_2_O_2_ consumption (Fig. 4h).

There have been extensive studies illustrating that the food vacuole of Para undergoes a period with acidic pH^30,32,45^, which, together with the H_2_O_2_ inside the vacuole, creates favorable conditions for the reaction between Fe (II) and H_2_O_2_ that yields •OH. We used 3′-(*p*-hydroxyphenyl) fluorescein (HPF) to track the formation of •OH. Interestingly, more aggregated green fluorescent vacuoles were observed in as-prepared E-Para (Fig. 4k) than in natural Para (Fig. 4j) at 0 h, verifying the rapid production of •OH inside the VSOs. The •OH production was then significantly enhanced in E-Para 8 hours post VSO engineering, indicating production of large amounts of •OH in the VSOs (Fig. 4i). Thereafter, the amount of •OH began to decline, probably due to the consumption of •OH during virus inactivation process. The colocalization of intracellular MNPs@Ab and the •OH fluorescence signal confirmed the crucial role of MNPs@Ab in the generation of •OH inside the vacuoles (Fig. 4k).

These phenomena indicated that the VSOs in E-Para resulted in a continuously higher level of •OH than native Para, leading to redox damage to the ingested virus. Together, the VSOs utilized synergistic interplay between MNPs@Ab and the vacuole environment to realize sustained production of •OH, which enabled efficient virus inactivation.

### Recyclability of E-para

**Fig. 5:**
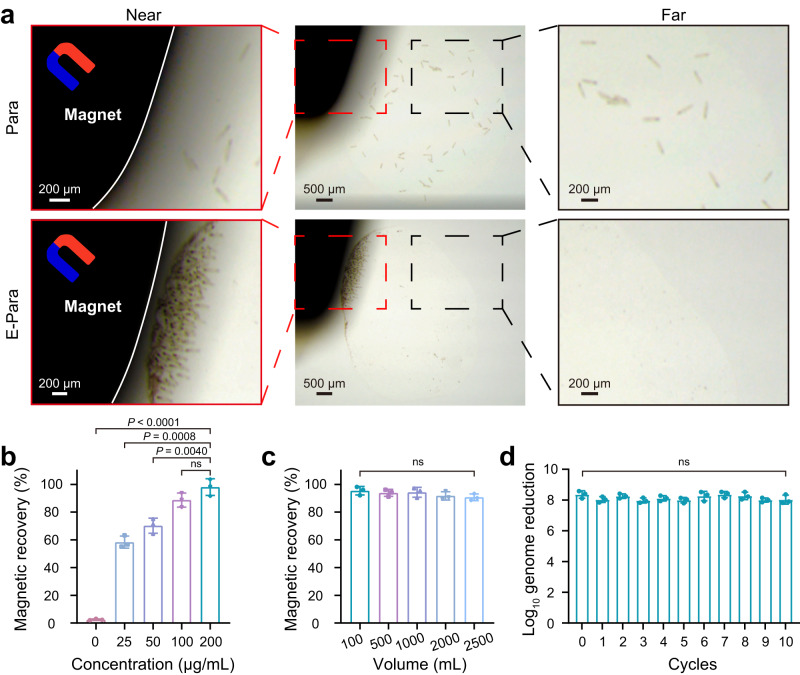
Magnetic directed recovery of E-Para. **a** Recovery of Para and E-Para with a magnet. The images were collected with a stereomicroscope. **b** Effect of incorporated MNPs@Ab concentration on magnetic recovery of the E-Para. The data are presented as the mean ± sd (*n* = 3). **c** Effect of solution volume on the magnetic recovery of E-Para. The data are presented as the mean ± sd (*n* = 3). **d** Reuse of E-Para. The data are presented as the mean ± sd (*n* = 3). In **b, c** and **d**, statistical significance was calculated via two-tailed Student’s t test. *P* < 0.05 was considered significant. Ns not significant.

In the case of biosecurity issues caused by virus-captured E-Para in the water, it is essential to recover the used E-Para after treatment. However, Para are difficult to collect by conventional collection methods due to their outstanding motility. The VSO-implanted E-Para was easily recovered from water solution by an external magnet (Fig. 5a) and returned to free movement after the magnet was removed (Supplementary Movie 3), confirming that magnetic recovery had no significant effect on the activity of E-Para. In addition, we killed E-Para by 4% paraformaldehyde and found that the killed E-Para showed superparamagnetic characteristic similar to that of MNPs@Ab, indicating that the killed E-Para is also recyclable because of VSO (Fig. 2h). The magnetic recovery efficiency of E-Para was related to the concentration of MNPs@Ab in VSO (Fig. 5b). In addition, magnetic recovery of E-Para from various volumes of solution was unaffected by the increased volume of water, indicating the availability of E-para removal without the need for extra treatment of the solution (Fig. 5c).

However, the magnetism variation of E-Para over time exhibited a slight decrease in the saturation magnetization from 3.4 to 1.7 emu/g within 24 h (Supplementary Fig. 18a). The Fe content in the E-Para kept declining within 72 h (Supplementary Fig. 19a), which led to the reduction of saturation magnetization. However, reduction of MNPs had negligible impact on the magnetic recovery rate of E-Para within 24 h (Supplementary Fig. 18b). In addition, when E-Para was cultured in starvation medium with time, their viability began to decline after 24 h (Supplementary Fig. 19b), indicating long-term toxicity of MNPs@Ab on Para. Proliferation of E-Para should be also restricted by using starvation medium to avoid the reduction of VSO caused by the division of E-Para (Supplementary Fig. 19c). Collectively, to avoid further loss of recyclability, the virus capture time should be limited to 24 h.

The recycled E-Para were then cultured in growth medium supplemented with *E.coli* to along the proliferation of Para (Supplementary Fig. 19d). To avoid the decrease of MNPs@Ab in individual cells (Supplementary Fig. 19c), proliferated Para were re-engineered with MNPs@Ab to restore the VSOs before reuse. The reactivated E-Para still resulted in an 8 log_10_ reduction of viral genome levels after ten cycles (Fig. 5d), indicating the feasibility of recycling and reusing this system. These results show that the magnetic VSOs enabled efficient recovery of E-Para with a magnetic field, which facilitated reuse and avoided the risk of infection, thus ensuring the environmental friendliness and biosafety of this strategy.

## Discussion

The key point of our design is to obtain an MNP-based biological virus-scavenging platform based on living Para. The incorporation of modified Fe_3_O_4_ magnetic nanoparticles into Para via food ingestion process is simple and efficient. Fe_3_O_4_ nanoparticles with proper modifiers shows biocompatibility in engineered Para at a reasonable concentration, and integrates with food vacuoles of Para to form a functionalized bioartificial organelle. The underlying mechanism of virus capture by E-Para is related to using MNPs@Ab to intervene the food ingestion, passage, and digestion process. First, the virus-scavenging food vacuoles organelles (VSOs) formed after feeding native Para with MNPs@Ab for 2 h via a food ingest pathway, by which the MNPs@Ab entered and retained inside food vacuoles for at least 24 h. In line with these results, previous report also shows that inorganic nanomaterials can be directly loaded into the vacuoles of Para through a ciliated groove^30^. The ingested nanomaterials such as quantum dots (QDs) halt the digestion and egestion of nanoparticle-containing vacuoles^46^, thus maintaining a long-life span inside the vacuoles. In addition, Para can accumulate and ingest food particles through their cytostomes and form vacuoles to circulate around the body along a definite path in endoplasm called cyclosis^30^. Thus, the VSOs with a long-life span can circulate in E-Para for at least 24 h, which is beneficial for the subsequent virus capture. Then, how does virus enter MNPs@Ab-containing VSOs? We observed dynamic membrane fusion and substance exchange between newly formed food vacuoles containing *E.coli* and circulating VSOs (Supplementary Fig. 13). Similarly, virus is also able to colocalize with MNPs@Ab. Together, these data verify that the virus can passage into the VSO of E-Para via fusion of new vacuoles with old vacuoles indicating a food passage pathway (Fig. 1c). After entering the VSO, the virus can be recognized and captured by the antibody-attaching MNPs. For native Para, virus cannot be efficiently retained inside food vacuoles and therefore excreted^22^. The use of difficult-to-discharge MNPs to deliver antibody into the food vesicles help to avoid rapid discharge of antibody and can thus retain and enrich antibody in the food vesicles. As such, virus capture efficiency of E-Para was significantly enhanced. We believe the virus-capture capacity of E-Para is mainly determined by the location and high local concentration of antibody. The vacuole-based ingestion of material particulates is common in single-celled protozoa and could be developed as a general modification strategy.

The advantage of using semiartificial organelles with peroxidase-like activity is that the hydrogen peroxide and the acidic environments in food vacuoles were stable and constant. A continuous supply of hydrogen peroxide significantly improved the catalytic effect of the MNPs, enabling nanozymes to produce •OH. Thus, the in vivo inactivation efficiency was significantly higher than that seen in the in vitro environment. Unlike other traditional disinfection methods, the VSO-bearing Para capture viruses and inactivates viruses with no need for additional disinfectant treatment. On the other hand, our result showed that native Para was able to kill 70.87% of the captured virus. Previous literature^22^ reports that Para inactivate virus either through production of metabolites that adversely affect the virus or by digesting the virus as food directly. As such, in addition to enhanced •OH production by MNPs@Ab, natural Para also killed virus by its intrinsic ability.

To avoid secondary pollution, we recover E-Para after incubation with virus for 24 h with a magnet and reused them again. The survival rate of E-Para was about 99% within 24 h, which ensures the effective virus capture and inactivation. Extending capture time is not recommended since the viability and superparamagnetic characteristic of E-Para reduced with time increasing, which increased the risk of virus leaking. In case that E-Para cannot be completely removed from water, the remained E-Para will not cause pollution of environmental water since Para is not a pathogenic pathogen and can even remove harmful microorganisms^19,29^ and pollutants^47,48^ from water environment. For drinking water purification, the remained E-Para in water may proliferate and become pollutants. However, the removal of Para is easier than the removal of waterborne virus, so the general purification procedure of drinking water can be used for purification.

The antibody-based VSO only captures virus that can be recognized by antibody. Nevertheless, antibody-based virus-specific strategy is not generic to other types of waterborne viruses. In case of purifying water of the broad variety of waterborne viruses, equipping the E-Para with VSO that target different types of potential viruses is required. Thus, we engineered the Para with MNPs@SA to target more potential viruses as SA is a well identified receptor for multiple viruses, such as enterovirus, influenza, and adenovirus. Para modified with MNPs@SA-containing VSO removes EV71, H1N1 and Ad5 by 99.99%, indicating the versatility of VSO. Either the antibody-based or SA-based VSOs achieved more than 4 log_10_ reductions in viral genome levels, exceeding the standard of WHO^38^.

As a proof-of-concept study, there is still considerable work needed before practical application. Based on our current understanding, several issues ought to be further addressed to ensure successful application, such as the cost and a broader-spectrum virus capture ability. In addition, investigations designed to evaluate the long-term biosafety of the E-Para-treated water are of crucial importance. The Para engineered by artificial organelles endows virus removal and inactivation with high efficiency, low energy consumption and minimal environmental pollution.

In summary, we designed a virus-scavenging semiartificial organelle to arm Para with the ability to capture the virus, deactivate the virus, and recover the captured virus. The customized VSOs are vacuole-derived compartments composed of virus-binding Fe_3_O_4_ magnetic nanoparticles, which circulate inside Para since the inorganic nanoparticles block the digestion and egestion of VSOs. The E-Para served as an efficient “microfactory” to inactivate the virus in situ through an enzyme-like catalysis pathway, by which the VSO produced large amounts of hydroxyl radicals to kill the captured viruses. Unlike conventional technologies that use high pressure filtration and detrimental chemical treatment, our strategy uses materials-engineered steerable microorganisms to collect and remove viruses, which requires minimal energy and is environmentally friendly. Overall, our study shows promise for functional modification of microorganisms by designing a nanotechnology-based artificial organelle, which is of considerable importance for the promotion of material-based biological evolution.

## Methods

### Materials

FeCl_3_·6H_2_O (99%), PEI (MW ~ 2500), PEG (MW ~ 2000) and PAA (MW ~ 3000) and sialic acid were purchased from Aladdin (Shanghai, China). Trisodium citrate dihydrate and BSA were purchased from MACKLIN (Shanghai, China). EDC, NHS, DMPO and Anti-Mouse EV71 Monoclonal Antibody were purchased from Merck (Darmstadt, Germany). Alexa Fluor 555 dye, Celltracker Green CMFDA and Goat Anti-Mouse IgG (H + L) Highly Cross-Adsorbed Secondary Antibody conjugated with Alexa Fluor Plus 555 were purchased from Thermo Fisher (Waltham, USA). A TIANamp Virus DNA/RNA Kit (#DP315) was purchased from Tiangen Biotech (Beijing, China). A One-Step TB Green PrimeScript^TM^ RT − PCR Kit II (#RR086A) was purchased from TaKaRa (Beijing, China). TMB single-component substrate solution (PR1200) was purchased from Solarbio (Beijing, China). SDS lysis was purchased from Beyotime (Shanghai, China). The ROSGreen^TM^ H_2_O_2_ probe was purchased from Maokang Biotechnology (Shanghai, China). HPF was purchased from AAT Bioquest (Sunnyvale, USA).

### Culture of para

*Paramecium caudatum* cultures were maintained in lettuce juice medium containing *E.coli*. as food. The preparation of lettuce juice medium was as follows^49,50^: fresh lettuce leaves were washed and immersed in boiling water for a few minutes and then placed in cold water to cool. Subsequently, the leaves were treated with the juicer repeatedly and squeezed out through gauze. For use as medium, the juice was diluted 1:40 with KDS buffer (2 mM C_6_H_5_Na_3_O_7_·2H_2_O, 0.6 mM KH_2_PO_4_, 1.4 mM Na_2_HPO_4_, 1.5 mM CaCl_2_) and incubated with *E.coli* for 24 h. Para were cultured in a constant temperature incubator at 25 °C.

### Synthesis of Fe_3_O_4_ magnetic nanoparticles (MNPs)

Briefly, FeCl_3_·6H_2_O (0.1 M) and trisodium citrate dihydrate (50 mM) were first dissolved in ethylene glycol (30 mL); afterward, NaAc (1.8 g) was added with stirring. The mixture was stirred vigorously for 30 min and then sealed in a Teflon-lined stainless-steel autoclave (50 mL capacity). The autoclave was heated at 200 °C, maintained for 10 h, and then allowed to cool to room temperature^51–53^. The black products were washed with ethanol and deionized water several times.

The synthesis of Fe_3_O_4_@PEI, Fe_3_O_4_@PEG, and Fe_3_O_4_@PAA was similar to that of MNPs, except that the stabilizer was PEI, PEG or PAA instead of sodium citrate.

### Preparation of Fe_3_O_4_ magnetic nanoparticles modified with a virus-targeted antibody (MNPs@Ab)

The MNPs@Ab was prepared by conjugating EV71 monoclonal antibody to the MNPs. The conjugation was realized through the *N*-ethyl-*N*′-(3-dimethylaminopropyl) carbodiimide (EDC)/*N*-hydroxysuccinimide (NHS) strategy^54,55^. In detail, 1 mL of MNPs (5 mg/mL) was mixed with EDC (0.1 M) and NHS (0.7 M) for 1 hour at room temperature. The remaining reagents in the coupling reaction were removed via a magnet. Subsequently, the nanoparticles were washed with phosphate-buffered saline (PBS) and finally resuspended in 1 mL of PBS. Next, 100 µl of EV71 antibody solution (1:100) was added to the activated nanoparticle suspension and incubated at room temperature for 2 h. Any excess unconjugated EV71 antibody was also removed via a magnet. BSA (1%) was used to block the nonspecific active sites.

### Construction of E-Para

Para were collected from lettuce juice medium containing *E.coli*, washed three times with KDS to remove the *E.coli* and then resuspended in KDS. Two hundred microliters of MNPs@Ab (1 mg/mL) was added to 800 μL Para solution (final concentration of cells: 8 × 10^3^ to 6.4 × 10^4^ cells/mL) to reach a final concentration of 200 μg/mL and then coincubated with Para for 2 h at 25 °C to construct E-Para. The E-Para were then collected via a magnet and resuspended in KDS before further processing.

### Viability of E-Para

Para were collected from growth medium and transferred to KDS buffer. Then, different concentrations of MNPs@Ab were added to Para culture medium to reach final concentrations of 100, 200, 400, 800, and 1600 μg/mL for 2 h. The obtained E-Para were then incubated for 24 h at 25 °C. Viable and nonviable cells were counted manually using a stereomicroscope (SZMN, SUNNY OPTICAL, China). Those Para that were immobile and did not preserve their typical shape were considered dead. Control experiments were performed using KDS buffer without any addition of MNPs@Ab. The survival rate (%) was calculated as follows^36^:

1 $${{{{{\rm{Survival\; rate}}}}}}\,(\%)=\frac{{N}_{2}}{{N}_{1}}\times 100\%$$

where *N*_*2*_ is the number of live Para or E-Para after incubation for 24 hours and *N*_*1*_ is the total number of Para or E-Para at the start of the experiment.

### Virus capture

E-Para (8 × 10^3^ to 6.4 × 10^4^ cells/mL) was incubated with virus solution (the volumes of the systems used in this study were 1 mL unless otherwise noted) for 24 h at 25 °C. Then, the E-Para that contained virus was removed by magnetic separation. The viral genome of virus solutions before and after treatment with E-Para were analyzed by RT‒qPCR assay. The primers used for RT‒qPCR are listed in Supplementary Table 2.

### Log reduction value

The log reduction value resulting from E-Para treatment was calculated as follows^56^:

2 $${{{{{\rm{Log}}}}}}\; {{{{{\rm{reduction}}}}}}\; {{{{{\rm{value}}}}}}={{{{{\rm{lg}}}}}}\frac{{I}_{0}}{{I}_{f}}$$

where *I*_*0*_ is the pretreatment concentration of the virus (copies/mL) and *I*_*f*_ is the post-treatment concentration of the virus.

### Fluorescence imaging

#### Fluorescence staining of EV71

One hundred microliters of virus solution was added to 500 μL of preprepared NaHCO_3_-Na_2_CO_3_ buffer solution (pH = 9.0), followed by 6 μL of AF555 dye solution dissolved in DMSO. The obtained mixed solution was injected into a dialysis bag and placed in normal saline for 48 h at 4 °C.

#### The fluorescence staining of E-Para and Para

Cell Tracker™ Green CMFDA dye solution (1 μL) was added to 1 mL concentrated E-Para solution. The obtained mixed solution was incubated for 30 min at 25 °C. The stained E-Para and Para were washed three times with KDS to remove excess dye.

The stained E-Para or Para were coincubated with stained virus for 4 h at 25 °C and fixed with paraformaldehyde (4%) for 12 h at 4 °C. The fluorescence images were collected by CLSM (BX61, Olympus, Japan) with FV1000-ASW Software (Version 4.1).

### Plaque-forming assay

The infectivity of EV71 in solution and in Para was assessed by plaque-forming assays in RD cells. For EV71 in solution, the virus solutions were diluted in PBS in a dilution series of 1:10. RD cells were seeded in a 12-well plate for 48 h, and then cells were infected with 1 mL of 10-fold viral dilutions for 1 h at 37 °C. For EV71 in E-Para and Para, the Para-containing virus was lysed at a concentration of 1:10 in SDS lysis buffer and KDS for 10 min to release the virus in Para, and the resulting virus solution was then diluted 10-fold to infect the RD cells for 1 h at 37 °C. The viral supernatants were replaced with DMEM containing low-melting-point agarose (1%) and FBS (2%). The cells were then incubated at room temperature for 20 min to solidify and then incubated at 37 °C for another 4–5 days. Cells were then fixed with formaldehyde (4%) for 30 min at room temperature, followed by staining with crystal violet solution (1%) for 15 min. Finally, all visible plaques were photographed and counted, and the final titers were calculated accordingly.

### In vitro •OH detection by EPR

•OH generated by the Fenton-like reaction between MNPs@Ab and H_2_O_2_ was detected by an EPR spectrometer (A300, Bruker, USA) with Bruker Biospin WinEPR Software (Version 4.40.11.65) at room temperature. 5,5-Dimethyl-1-pyrroline-*N*-oxide (DMPO) was used as a spin trap for the detection of •OH. Then, 100 μL of DMPO (0.15 M) was added to 50 μL of MNPs@Ab solution and detected immediately after the addition of 50 μL of H_2_O_2_ (10 M). H_2_O_2_ and MNPs@Ab only were used as controls. The settings of the EPR measurement parameters were as follows: 20.5 mW microwave power, 120 G scan range and 2 G amplitude modulation.

### Peroxidase-like activity of E-Para

#### Qualitative analysis

One hundred microliters of TMB single-component substrate solution was added to 900 μL of KDS containing Para (8 × 10^3^ cells/mL). The mixture of TMB and E-Para was incubated for 20 min at 25 °C in the dark. The photographs were collected with an inverted fluorescence microscope (IX73, Olympus, Japan).

#### Quantitative analysis

One hundred microliters of TMB single-component substrate solution was added to 900 μL of KDS containing Para (8 × 10^3^ cells/mL) or 900 μL of MNPs@Ab solution (200 μg/mL). The mixture of TMB and E-Para was incubated for 20 min at 25 °C in the dark, and then 100 μL of SDS lysis buffer was added and incubated for another 10 min. Then, 100 μL of the above mixed solution was added to a 96-well plate, and the absorbance at 652 nm was measured by a microplate reader (Synergy H1, BioTek, USA) with Gen5 Software (Version 3.08). Three parallel groups were set up for each sample.

### In vivo H_2_O_2_ measurement

#### Qualitative analysis

The intracellular H_2_O_2_ levels of E-Para and Para were measured by ROSGreen™. Briefly, 500 μL of ROSGreen™ H_2_O_2_ Probe (10 μΜ) was added to 500 μL of KDS containing Para (8 × 10^3^ cells/mL) and incubated for 30 min at room temperature in the dark. After that, the Para were fixed with paraformaldehyde (4%) for 1 h at room temperature, washed three times with KDS and observed by the FITC channel of an inverted fluorescence microscope (IX73, Olympus, Japan).

#### Quantitative analysis

Fifty microliters of ROSGreen™ H_2_O_2_ Probe (10 μΜ) was added to 50 μL of KDS containing Para (8 × 10^3^ cells/mL) and incubated in a black 96-well plate with a clear bottom for 30 min at room temperature in the dark. Four parallel groups were set up for each sample. The fluorescence intensity was measured and recorded by means of a fluorescence microplate reader (Synergy H1, BioTek, USA) with Gen5 Software (Version 3.08) at 490 nm excitation and 525 nm emission. KDS was used as a control. The intracellular H_2_O_2_ level was calculated according to the fluorescence value and standard curve of H_2_O_2_ (Supplementary Fig. 20).

### In vivo •OH measurement

#### Qualitative analysis

The intracellular •OH levels of E-Para and Para were measured by HPF. Briefly, 500 μL of an HPF working solution (20 μΜ) was added to 500 μL of KDS containing Para (8 × 10^3^ cells/mL) and incubated for 45 min at room temperature in the dark. After that, the E-Para and Para were fixed with paraformaldehyde (4%) for 1 h at room temperature and washed three times with KDS. Then, the E-Para and Para were observed by the FITC channel of an inverted fluorescence microscope (IX73, Olympus, Japan).

#### Quantitative analysis

The fluorescence intensity of HPF was measured by ImageJ Software (Version 1.51j8, USA).

### Magnetic recovery

The E-Para containing virus was collected with a magnet. The number of E-Para collected by magnetic force and the total number of E-Para were recorded manually with a stereomicroscope (SZMN, SUNNY OPTICAL, China). The magnetic recovery efficiency of E-Para was calculated according to the following formula:

3 $${{{{{\rm{Magnetic}}}}}}\; {{{{{\rm{recovery}}}}}}\; {{{{{\rm{efficiency}}}}}}\,(\%)=\frac{{M}_{2}}{{M}_{1}}\times 100\%$$

where *M*_*2*_ is the number of E-Para collected by magnetic force and *M*_*1*_ is the total number of Para.

### Reuse of E-Para

The recovered E-Para were cultured for 24 h in a feeding medium containing *E.coli*. Then, these recovered E-Para were incubated with MNPs@Ab (200 μg/mL) for another 2 h to develop VSOs and released to treat 1 mL of virus-containing water ( ~ 10^8^ copies/mL) again. The virus genome remaining after treatment of E-Para was measured by RT-qPCR, and the Log_10_ genome reduction value was calculated.

### Statistics and reproducibility

Statistical significance was evaluated using unpaired two-tailed Student’s t test or two-way analyses of variance (ANOVA) with Tukey’s multiple-comparison test as described in the figure legends. Three independent experiments were repeated to obtain the representative images (Figs. 2b, d–g, 3a, b, 4g, j, k; and Supplementary Fig. 8-10, Supplementary Fig. 13a–h, Supplementary Fig. 17a, b, Supplementary Fig. 19c).

### Reporting summary

Further information on research design is available in the Nature Portfolio Reporting Summary linked to this article.

### Supplementary information

Supplementary Information

Reporting Summary

Description of Additional Supplementary Files

Supplementary Movie 1

Supplementary Movie 2

Supplementary Movie 3

### Source data

Source Data

## Data Availability

The source data generated in this study are provided in the Source Data file. The full image dataset is available from the corresponding authors upon request. Source data are provided with this paper.
