# Supplementary material for: Material-engineered bioartificial microorganisms enabling efficient scavenging of waterborne viruses
Source: Nat Commun. 2023 Aug 3;14:4658. doi: 10.1038/s41467-023-40397-5 (PMC10400550; doi:10.1038/s41467-023-40397-5)
Supplement: Supplementary file 1 — Supplementary Information [file 41467_2023_40397_MOESM1_ESM.pdf]

Supplementary Information

Material-Engineered Bioartificial Microorganisms Enabling Efficient Scavenging of Waterborne Viruses

Huixin Li[1,2,3,4], Yanpeng Xu[5], Yang Wang[6], Yihao Cui[1], Jiake Lin[1], Yuemin Zhou[1], Shuling Tang[1], Ying Zhang[1,2,3,4], Haibin Hao[1,2,3], Zihao Nie[2,3,4], Xiaoyu Wang[2,3,4,*] and Ruikang Tang[1,2,3,*]

[1]Department of Chemistry, Zhejiang University, Hangzhou, Zhejiang, China

[2]Department of Cardiology, Sir Run Run Shaw Hospital, School of Medicine, Zhejiang University, Hangzhou, Zhejiang, China

[3]Qiushi Academy for Advanced Studies, Zhejiang University, Hangzhou, Zhejiang, China

[4]Key Laboratory of Cardiovascular Intervention and Regenerative Medicine of Zhejiang Province, Hangzhou, Zhejiang, China

[5]Laboratory of Virology, Beijing Key Laboratory of Etiology of Viral Diseases in Children, Capital Institute of Pediatrics, Beijing, China

[6]School of Chemistry and Chemical Engineering, Nantong University, Nantong, Jiangsu, China

*Corresponding authors: xy_wang@zju.edu.cn (Xiaoyu Wang) and rtang@zju.edu.cn (Ruikang Tang)

Supplementary Methods

Enterovirus 71 preparation

Enterovirus 71 (EV71) was produced and titrated in rhabdomyosarcoma (RD) cells. RD cells (Catalog number: CTCC-400-0203) were purchased from Meisen Chinese Tissue Culture Collections (Zhejiang, China). The RD cells were infected with EV71 for 48 hours, and the virus was harvested by freeze–thaw cycles. All virus stocks were stored at -80 °C until use.

Enzyme-linked immunosorbent assay (ELISA)

We used ELISA assay to determine if modification of anti-EV-71 antibodies with MNPs enabled virus binding by the MNPs@Ab. A 100 µL solution of EV71 (10^6 PFU/mL) in coating buffer (0.05 M $NaHCO_3$-Na_2CO_3 buffer, pH=9.6, 0.22 µm filtered) was coated on a 96-well polystyrene microplate overnight at 4 °C and then washed three times with 100 µL of PBS (0.01 M, pH=7.4). Then, the plate was blocked with 100 µL of blocking solution (5% BSA), incubated for 1 hour at 37 °C and washed three times with PBS. Afterward, a series of concentrations of MNPs and MNPs@Ab were added to the wells (100 µL) and incubated for 1 hour at 37 °C to allow interactions with the EV71. After incubation, chromogenic agent TMB and H_2O_2 were directly added to the wells and incubated at 25 °C for 15 min. Due to the peroxidase activity of the MNPs, the MNPs@Ab bound to the EV71 developed a blue color. After 15 min, 100 µL of 10% H_2SO_4 solution was added to stop the reaction, followed by measurement of absorbance at 450 nm using an ELISA reader (Synergy H1, Biotek, USA). MNPs that were bound specifically to EV71 remained in the wells for color development, while those that did not bind to EV71 were removed during cleaning and thus did not produce color.

Magnetometry

Paraformaldehyde (4%)-fixed Para was prefrozen at -80 °C for 12 hours and then processed in a Freeze Dry System (Labconco, USA) for 24 hours. The hysteresis loop of fixed samples was analyzed using a Magnetic Property Measurement System (MPMS) (Quantum Design, USA). Field-dependent magnetization curves were measured at 300 K as a function of the external field.

Electron microscopy imaging

The suspension containing MNPs@Ab was dropped onto carbon-coated copper TEM grids and then dried at room temperature. The observations were performed using TEM (JEM-1230, Jeol, Japan).

The Para samples were first fixed with glutaraldehyde (2.5%) in KDS buffer (0.1 M, pH 7.0) for more than 4 hours, washed three times in phosphate buffer (0.1 M, pH 7.0) for 15 min at each step, postfixed with OsO_4 (1%) in phosphate buffer for 1-2 hours and washed three times in phosphate buffer (0.1 M, pH 7.0) for 15 min at each step. Then, the samples were first dehydrated by a graded series of ethanol (30%, 50%, 70%, 80%) for approximately 15 min at each step and then dehydrated by a graded series of acetone (90%, 95%) for approximately 15 min at each step. Finally, the samples were dehydrated twice with absolute acetone for 20 min each. The specimens were placed in a 1:1 mixture of absolute acetone and the final Spurr resin mixture for 1 hour at room temperature and then transferred to a 1:3 mixture of absolute acetone and the final resin mixture for 3 hours and to the final Spurr resin mixture overnight. The specimens were placed in Eppendorf tubes containing Spurr resin and heated at 70 °C for more than 9 hours. The specimens were sectioned in a LEICA EM UC7 ultratome, and sections were stained with uranyl acetate and alkaline lead citrate for 5 to 10 min and observed by TEM (JEM-1230, Jeol, Japan). The above processing process was completed in the Bio-ultrastructure Analysis Lab of Analysis Center of Agrobiology and Environmental Sciences, Zhejiang University.

Intracellular iron quantification

A NexION 300X ICP–MS (PerkinElmer, USA) was employed to determine the iron content in E-Para. *Paramecium* samples were digested overnight in 1 mL of concentrated nitric acid and then bathed in 90 °C water for 2 hours. The solutions were then diluted in ultrapure water and filtered through a 0.22 μm filter for subsequent analysis. The radio frequency was 1300 W; the plasma gas flow rate was 17.0 L/min; the auxiliary gas flow rate was 1.2 L/min; the nebulizer gas flow rate was 1.0 L/min; and the dwell time was 50 ms. Calibration standards were provided by the Analysis Center of Agrobiology and Environmental Sciences, Zhejiang University.

Virus capture capacity of different material and *Paramecium*

For MNPs and MNPs@Ab: MNPs and MNPs@Ab (40 μg) were incubated with 1 mL of virus solution (2.48×10^5 copies/mL) for 24 hours at 25 °C. This concentration of the materials was roughly equivalent with the MNPs@Ab content in E-Para. Then, MNPs and MNPs@Ab was removed by magnetic separation. The viral genome levels of the virus solutions before and after treatment with MNPs or MNPs@Ab were analyzed by RT–qPCR assay.

For Para and E-Para: Para and E-Para (8×10^3 cells) were incubated with 1 mL of virus solution (2.48×10^5 copies/mL) for 24 hours at 25 °C. Then, the Para and E-Para was removed from the solution. The viral genome levels of the virus solutions before and after treatment with Para or E-Para were analyzed by RT–qPCR assay.

For Para-MNPs: Similar to the preparation of E-Para, Para was engineered by MNPs to prepare Para-MNPs. The concentration of the used MNPs was 200 μg/mL. The virus capture process by Para-MNPs is similar to that by E-Para mentioned above.

For Para-Ab: Para was fed with antibody to prepare Para-Ab. Two hundred microliters of 1:12500 EV71 monoclonal antibody was added to 800 μL Para solution (final concentration of cells: 8×10^3 cells/mL) and then incubated with Para for 2 hours at 25 °C to construct Para-Ab. The concentration of the antibody was roughly equivalent with the antibody content on MNPs@Ab used in the construction of E-Para. The virus capture process by Para-Ab is similar to that by Para mentioned above.

Cell number-dependent virus capture

For an initial EV71 concentration of 1.5×10^5 copies/mL: E-Para (2×10^3, 4×10^3, 8×10^3, 1.6×10^4, 3.2×10^4 cells) were incubated with 1 mL of the virus solution for 24 hours at 25 °C. Then, the E-Para that contained virus was removed by magnetic separation. The viral genome levels of the virus solutions before and after treatment with E-Para were analyzed by RT–qPCR assay.

For an initial EV71 concentration of 3.2×10^8 copies/mL: E-Para (4×10^3, 8×10^3, 1.6×10^4, 3.2×10^4, 6.4×10^4, 8×10^4 cells) were incubated with 1 mL of virus solution for 24 hours at 25 °C. Then, the E-Para that contained virus was removed by magnetic separation. The viral genome levels of the virus solutions before and after treatment with E-Para were analyzed by RT–qPCR assay.

Virus capture with different volumes

E-Para (6.4×10^4 cells/mL) were incubated with different volumes (100, 500, 1000, 2000, 2500 mL) of virus solution with an initial concentration of ~10^8 copies/mL for 24 hours at 25 °C. Then, the E-Para that contained virus was removed by magnetic separation. The viral genome levels of the virus solutions before and after treatment with E-Para were analyzed by RT–qPCR assay and the log_{10} genome reduction was calculated.

Synthesis of MNPs@SA

The method for synthesis of MNPs@SA was similar to that use for Fe_3O_4 magnetic nanoparticles. $FeCl_3·6H_2O$ (0.1 M) and sialic acid (50 mM) were first dissolved in ethylene glycol (30 mL); afterward, NaAc (1.8 g) was added with stirring. The mixture was stirred vigorously for 30 min and then sealed in a Teflon-lined stainless-steel autoclave (50 mL capacity). The autoclave was heated at 200 °C, maintained there for 10 hours, and then allowed to cool to room temperature. The black product was washed with ethanol and deionized water several times.

Construction of E-Para-SA

Para were collected from lettuce juice medium containing *Escherichia coli* (*E.coli*), washed three times with KDS to remove the *E.coli* and then resuspended in KDS. Two hundred microliters of MNPs@SA (1 mg/mL) was added to 800 µL of Para solution (final concentration of cells: 6.4×10^4 cells/mL) to reach a final concentration of 200 µg/mL and then coincubated with Para for 2 hours at 25 °C to construct E-Para-SA. The E-Para-SA were then collected via a magnet and resuspended in KDS before further processing.

Virus capture by E-Para-SA.

E-Para-SA (6.4×10^4 cells/mL) were incubated with 1 mL of virus mixture containing EV71 (8.2×10^7 copies/mL), H1N1(1.4×10^8 copies/mL) and Ad5 (4.6×10^7 copies/mL) for 24 hours at 25 °C. Then, the E-Para-SA that contained virus was removed by magnetic separation. The viral genome levels of the virus mixture after treatment with E-Para-SA and Para were analyzed by RT–qPCR.

RNA isolation and RT–qPCR

Total viral RNA was extracted using a TIANamp Virus DNA/RNA Kit according to the manufacturer's instructions and then quantified by one−step quantitative real−time RT−qPCR using a One Step TB Green PrimeScript™ RT−PCR Kit II according to the manufacturer's protocol with specific primers.

Quantitative RT–qPCR was performed using an Applied Biosystems 7500 Fast Real-Time PCR System (ThermoFisher, USA). The RT–qPCR was applied to 20 µL systems (10 µL of 2X One Step TB Green RT–qPCR Buffer 4, 0.8 µL of PrimeScript 1 Step Enzyme Mix 2, 0.8 µL each of forward and reverse primers (10 µM), 0.4 µL of ROX Reference Dye II (50X)*3, 2 µL of total RNA, and 5.2 µL of RNase Free dH_2O). RT–qPCR response procedures (Stage 1: 1 cycle at 42 °C for 5 min; Stage 2: 95 °C for 10 s; Stage 3: 40 cycles at 95 °C for 3 s, 60 °C for 30 s; Stage 4: 1 cycle at 95 °C for 15

s, 60 °C for 1 min, 95 °C for 15 s) were applied according to the manufacturer's instructions. The primers used for RT–qPCR are listed in Supplementary Table 2[1,2].

Indirect immunofluorescence imaging

EV71 (10^3 PFU/mL) in DMEM was mixed with Para and E-Para and cocultured for 24 hours at 25 °C. Para was recovered by a magnet, and the supernatant was diluted with DMEM containing 2% FBS to infect RD cells seeded in 35 mm cell culture glass-bottomed dishes for 1 hour. The infected cells were washed three times with phosphate-buffered saline (PBS) and then cultured in DMEM containing 2% FBS. After 24 hours of infection, the cells were fixed with precooled acetone at -20 °C for 30 min. The cells were then washed with PBS three times and blocked with 5% BSA solution for 1 hour at 37 °C. Next, the cells were washed with PBS and then incubated with Anti-Mouse EV71 Monoclonal Antibody (MAB979, Merck, 1:1000) for 1 hour at 37 °C; the cells were then washed with PBS and incubated with goat anti-mouse IgG (H+L) Highly Cross-Adsorbed Secondary Antibody conjugated with Alexa Fluor Plus 555 (10 μg/mL) for 1 hour at 37 °C. After washing the infected cells with PBS, DAPI (Beyotime, China) was added, and the cells were incubated at room temperature for 15 min to stain the nuclei. The cells were observed with an inverted fluorescence microscope (IX73, Olympus, Japan) after washing with PBS.

Calculation of inactivation efficiency

The inactivation efficiency (P, %) was calculated as follows:

$$\text{Inactivation efficiency (\%)}=\frac{P_1}{P_2}\times 100\% \qquad (1)$$

where P_1 is the infectivity of the virus released from E-Para and P_2 is the infectivity of the virus captured by E-Para.

P_2 was calculated as follows:

$$P_2=P_3- P_4 \qquad (2)$$

where P_3 is the infectivity of the original virus solution and P_4 is the infectivity of the virus solution after treatment with E-Para.

Remaining viral genome in supernatant and inside E-Para/Para

E-Para (8×10^3 cells/mL) were incubated with 1 mL of virus solution (10^5 copies/mL) for 24 hours at 25 °C. Then, the E-Para that contained virus was removed by magnetic separation. The viral genome levels of the virus solutions after treatment with E-Para or Para were analyzed by RT–qPCR assay. For EV71 inside E-Para and Para, the Para-containing virus was lysed at a concentration of 1:10 in SDS lysis buffer and KDS for 10 min to release the virus in the Para, and the viral genome level in the resulting virus solution was measured by RT–qPCR assay.

Supplementary Notes

The main processes of Fenton reaction[3,4,5]:

$$Fe^{2+}+H_2O_2 \rightarrow Fe^{3+}+OH^-+\bullet OH$$

$$Fe^{3+}+H_2O_2 \rightarrow Fe^{2+}+HO_2^{\bullet}+H^+$$

Guide for E-Para fabrication and storage in daily life

Para is sold as fish feed. The obtained Para can store in a four-degree refrigerator for a month.

When needed, the stored Para can be revived by adding culture medium and incubating at room temperature. The culture of Para in the laboratory was described in the main text. The cultivation of Para in daily life is not difficult when there is no laboratory equipment. For example, *Paramecium* can be harvested from ponds. Yeast and straw culture can be used as food for *Paramecium*. Of note, *Paramecium* should be incubated at room temperature. Temperatures that are too high (higher than 35 °C) or too low (lower than 10 °C) may affect the survival rate of Para.

The engineering of Para was described in the main text. Surface modification methods for Fe_3O_4 nanoparticles are well established and antibody or sialic acid modified nanoparticles can be purchased from companies such as Xianfeng Nano (China). The engineering of Para in daily life can be realized by incubating the above-mentioned commercial Fe_3O_4 nanoparticles with Para at room temperature for 2 hours. The obtained E-Para is available for scavenging virus in the water. For 2 L water, at least 1.6×10^7 Para are needed. Since E-Para are usually prepared before use, they cannot be stored for long periods of time.

Supplementary Figures

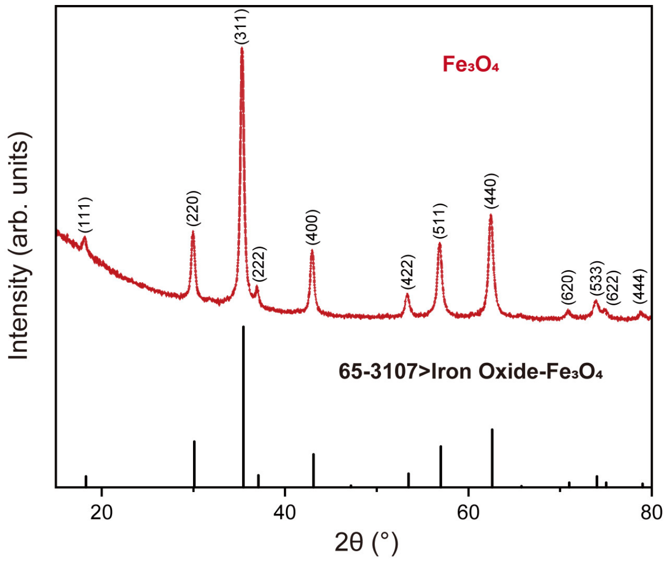

Supplementary Figure 1. Comparison of pXRD data for MNPs and the Fe_3O_4 standard. The diffraction peak positions and intensities for the synthesized MNPs were in good agreement with the reference data for the Fe_3O_4 standard (JCPDS card No. 65-3107).

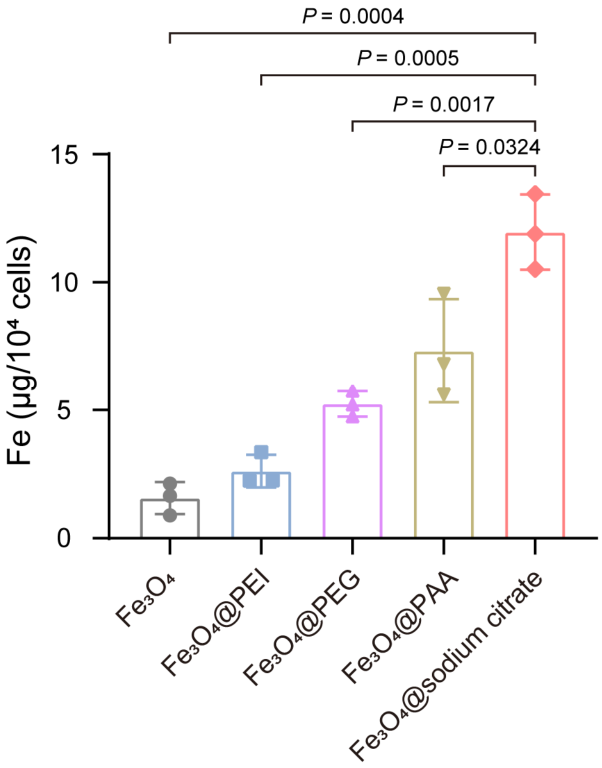

Supplementary Figure 2. Stabilities of Fe_3O_4 with different surface modifications inside Para. Para was incubated with Fe_3O_4 nanoparticles with different surface modifications (none, PEI, PEG, PAA, sodium citrate) for 2 hours, and then diverse engineered Para samples were obtained. The Fe content in the engineered Para was measured after 24 hours to evaluate the stability of the materials. The concentration of the materials was 200 μg/mL. The data are presented as the mean ± s.d. (n = 3). Statistical significance was calculated via two-tailed Student's t test. $P < 0.05$ was considered significant. Ns, not significant.

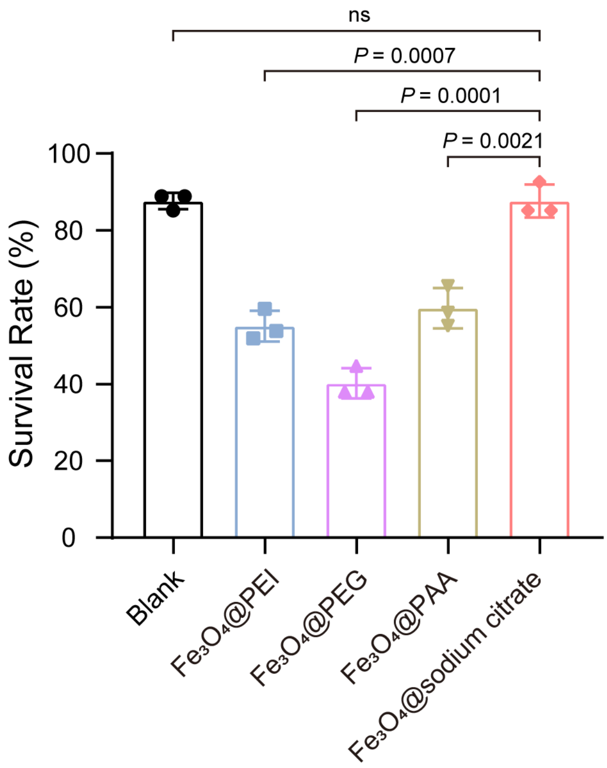

Supplementary Figure 3. Cytotoxicity of Fe_3O_4 with different surface modifications to Para. Para was incubated with Fe_3O_4 nanoparticles with different surface modifications (none, PEI, PEG, PAA, sodium citrate) for 24 hours, and the survival rate was calculated to evaluate the cytotoxicity of the materials. The blank group represents the survival rate of Para without any addition of Fe_3O_4. The concentration of the materials was 200 μg/mL. The data are presented as the mean ± s.d. (n = 3). Statistical significance was calculated via two-tailed Student's t test. $P < 0.05$ was considered significant. Ns, not significant.

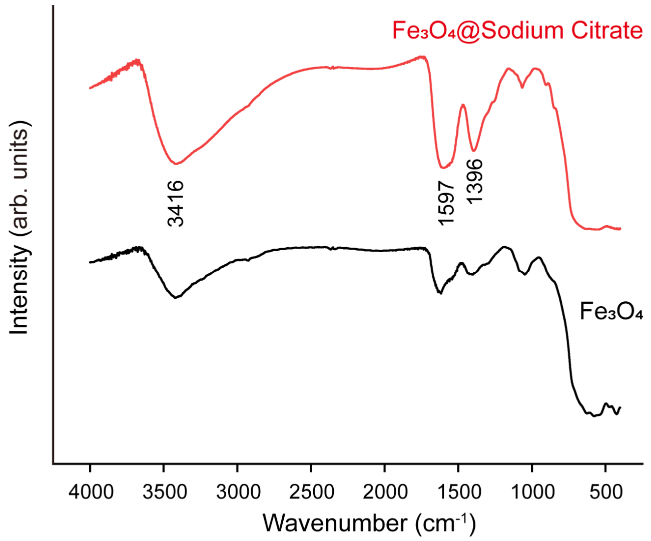

Supplementary Figure 4. FTIR analysis. Fe_3O_4@sodium citrate and Fe_3O_4 were vacuum dried at room temperature, collected and subjected to FTIR spectroscopy (NICOLET iS50FT-IR, Thermo Fisher, USA).

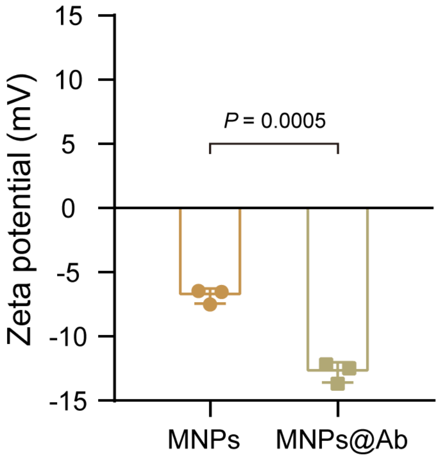

Supplementary Figure 5. Zeta potential of MNPs and MNPs@Ab. MNPs are magnetic ferric oxide nanoparticles with sodium citrate as a stabilizer and MNPs@Ab are antibody-modified MNPs. The data are presented as the mean ± s.d. (n = 3) Statistical significance was calculated via two-tailed Student's t test. $P < 0.05$ was considered significant. Ns, not significant.

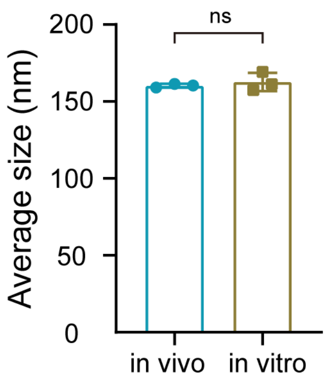

Supplementary Figure 6. Size analyses of MNPs@Ab in E-Para and in vitro. The average size of MNPs@Ab particles in E-Para was approximately 160.32 nm and that in vitro was approximately 162.67 nm. The *P* value showed that there was no significant difference between the two sizes. The data are presented as the mean ± s.d. (n = 3). Statistical significance was calculated via two-tailed Student's t test. $P < 0.05$ was considered significant. Ns, not significant.

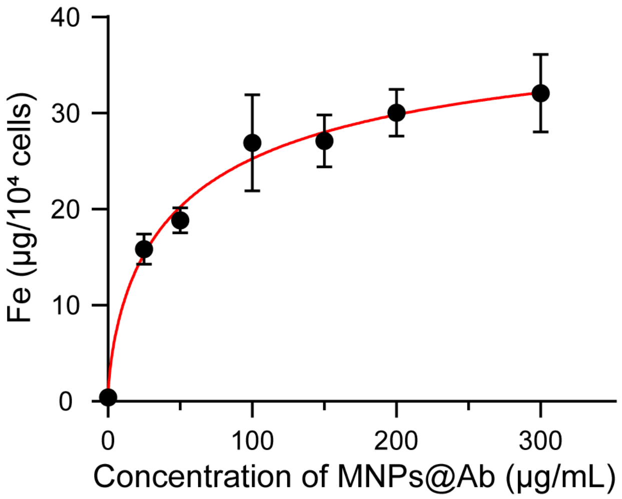

Supplementary Figure 7. Dose dependence of Fe in E-Para on MNPs@Ab. Fe inside E-Para after coincubation with a gradient series concentration of MNPs@Ab for 2 hours. The Fe concentration in Para was approximately 0.413 ± 0.007 μg/10^4 cells. The data are presented as the mean ± s.d. (n = 3).

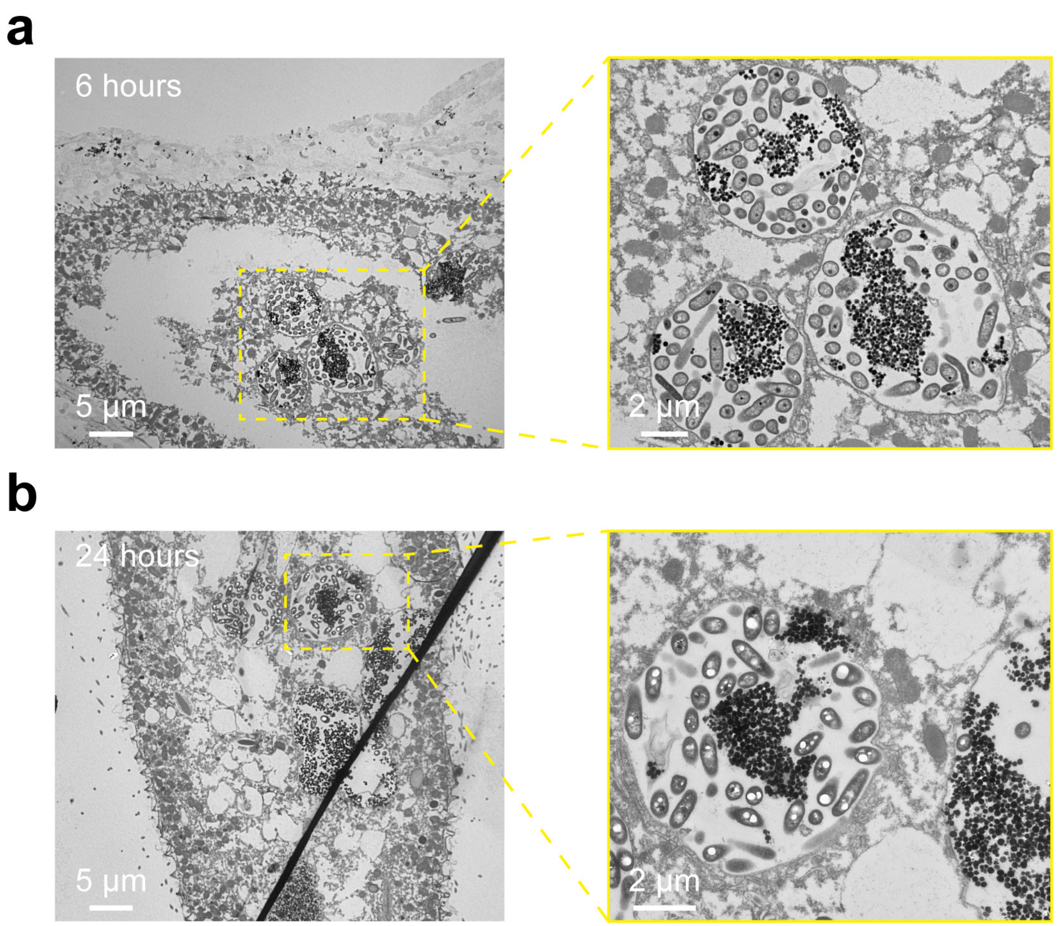

Supplementary Figure 8. Stability of VSO in E-Para. a TEM image of VSO inside E-Para after 6 hours. **b** TEM image of VSO inside E-Para after 24 hours.

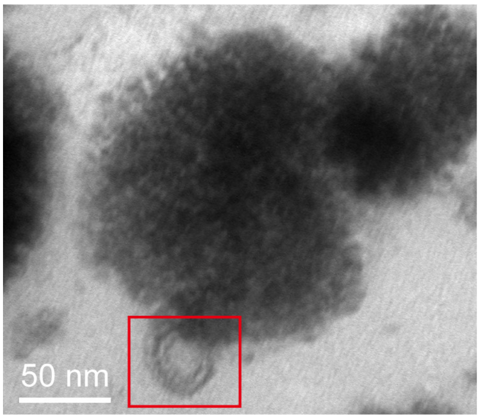

Supplementary Figure 9. Specific adsorption of MNPs@Ab inside E-Para to ingested viruses. E-Para was incubated with EV71 for 4 hours, and the TEM image showed that the ingested virus was tightly attached to the surface of MNPs@Ab.

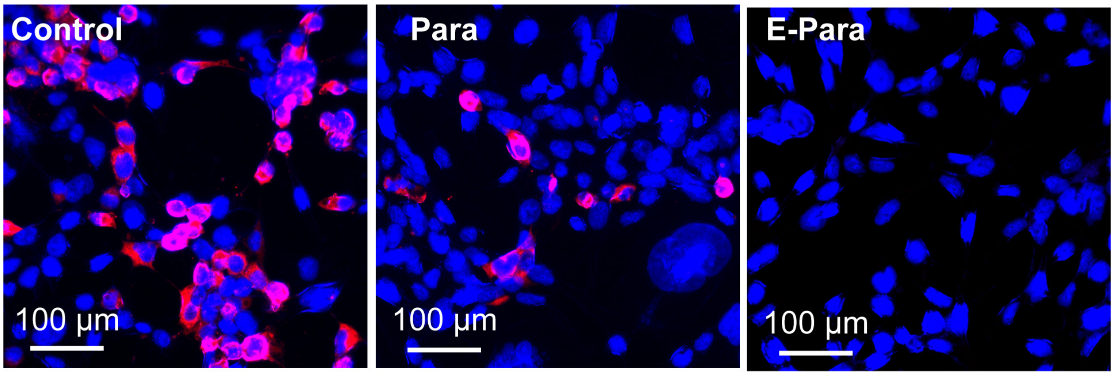

Supplementary Figure 10. IFA results for remaining EV71 after treatment with Para or E-Para. The nucleus was stained with DAPI (blue), and the infected RD cells were incubated with EV71 antibody and then stained with secondary antibody conjugated with Alexa Fluor Plus 555 (red).

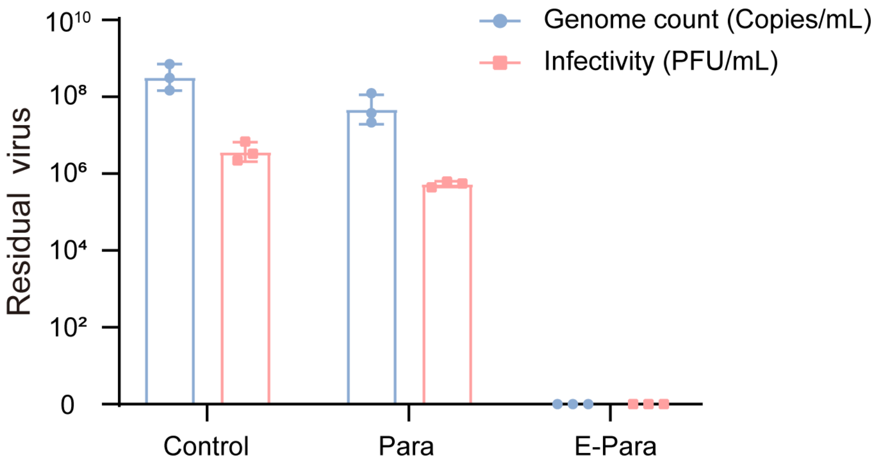

Supplementary Figure 11. Genome count and infectivity for the total amount of virus after treatments with E-Para and Para (6.4×10^4 cells/mL). The data are presented as the mean ± s.d. (n = 3).

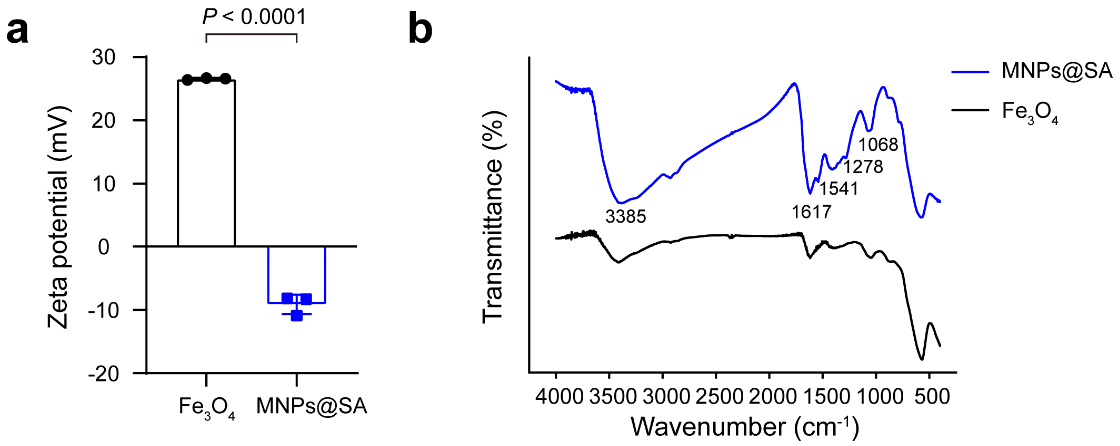

Supplementary Figure 12. Characterization of MNPs@SA. a Zeta potential of MNPs@SA. The zeta potentials of MNPs@SA and Fe_3O_4 and were -9.1 mV and 26.6 mV, respectively. The data are presented as the mean ± s.d. (n = 3). Statistical significance was calculated via two-tailed Student's t test. $P < 0.05$ was considered significant. Ns, not significant. **b** FTIR analysis of MNPs@SA. The Fe_3O_4 and MNPs@SA were vacuum dried at room temperature, collected and examined with FTIR spectroscopy (NICOLET iS50FT-IR, Thermo Fisher, USA).

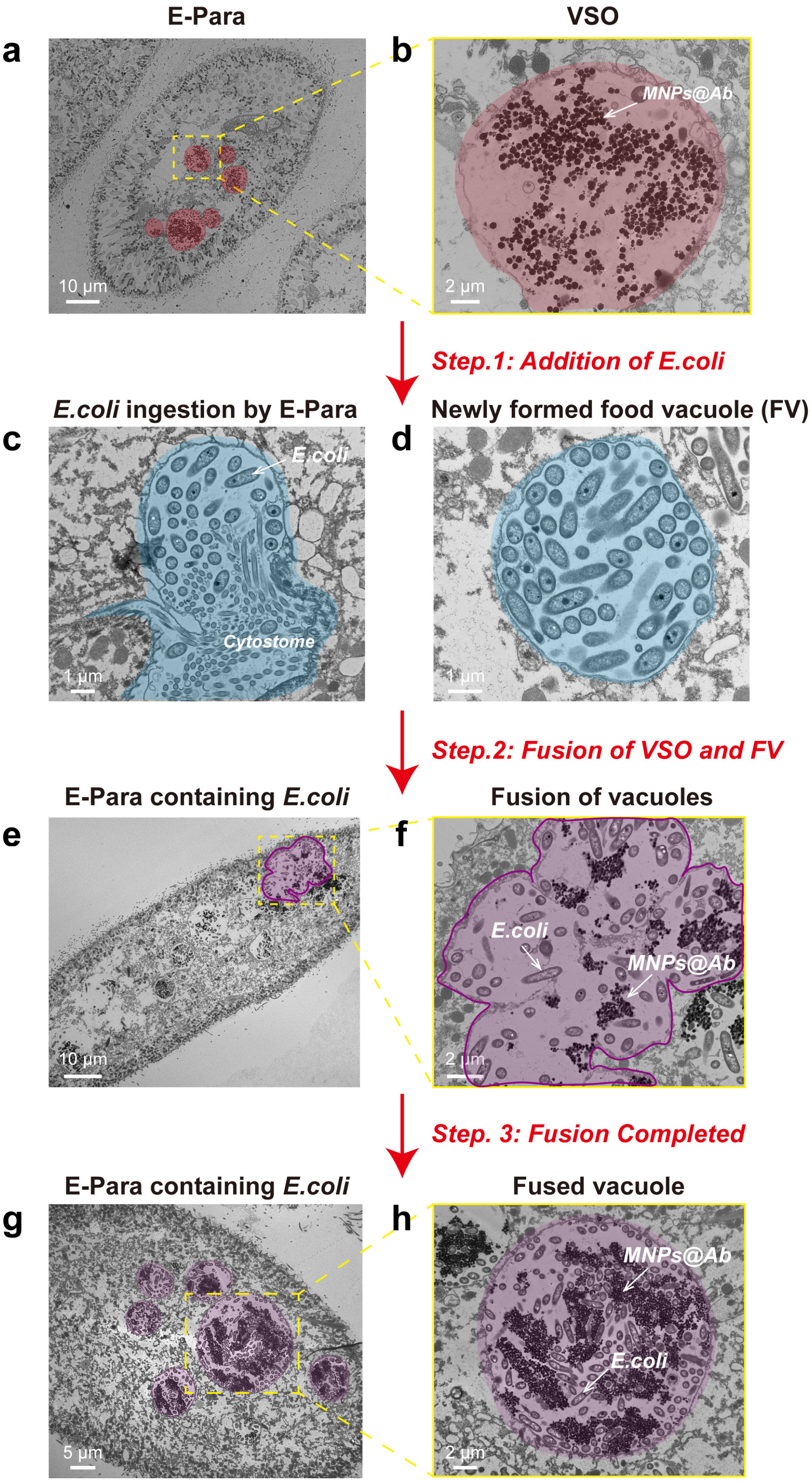
E-Para
VSO
a
b
MNPs@Ab
10 μm
2 μm
Step.1: Addition of E.coli
E.coli ingestion by E-Para
Newly formed food vacuole (FV)
c
d
E.coli
Cytostome
1 μm
1 μm
Step.2: Fusion of VSO and FV
E-Para containing E.coli
Fusion of vacuoles
e
f
E.coli
MNPs@Ab
10 μm
2 μm
Step. 3: Fusion Completed
E-Para containing E.coli
Fused vacuole
g
h
MNPs@Ab
E.coli
5 μm
2 μm

Supplementary Figure 13. Mechanism of food vacuole fusion in E-Para. a TEM image of E-Para. **b** VSO in E-Para. Large amounts of MNPs@Ab were observed in VSO. **c** *E.coli* ingestion by E-Para. **d** Newly formed food vacuole (FV) in E-Para after ingestion of *E.coli*. Large amounts of *E.coli* were observed in FV. **e** E-Para containing *E.coli* in which the vacuoles were fusing. **f** Fusion of the vacuoles in E-Para. The VSO and FV were fused with each other. **g** E-Para containing *E.coli* in which the vacuoles have been fused. **h** Fused vacuole where MNPs@Ab and *E.coli* were co-located.

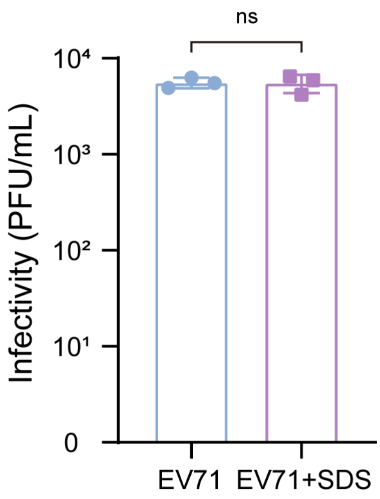

Supplementary Figure 14. Effect of SDS lysis buffer on the infectivity of EV71. The infectivity of EV71 was tested by plaque-forming assays after adding SDS to the virus solution (1:10) for 10 min. EV71 without treatment was used as a control. The data are presented as the mean ± s.d. (n = 3). Statistical significance was calculated via two-tailed Student's t test. $P < 0.05$ was considered significant. Ns, not significant.

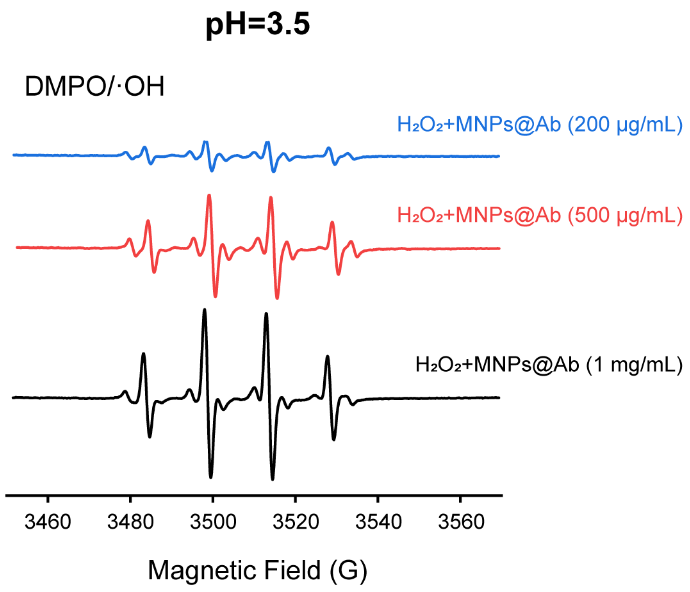

Supplementary Figure 15. EPR spectrum of H_2O_2 with different concentrations of MNPs@Ab. At pH 3.5, the •OH signal increased with increasing concentration of MNPs@Ab.

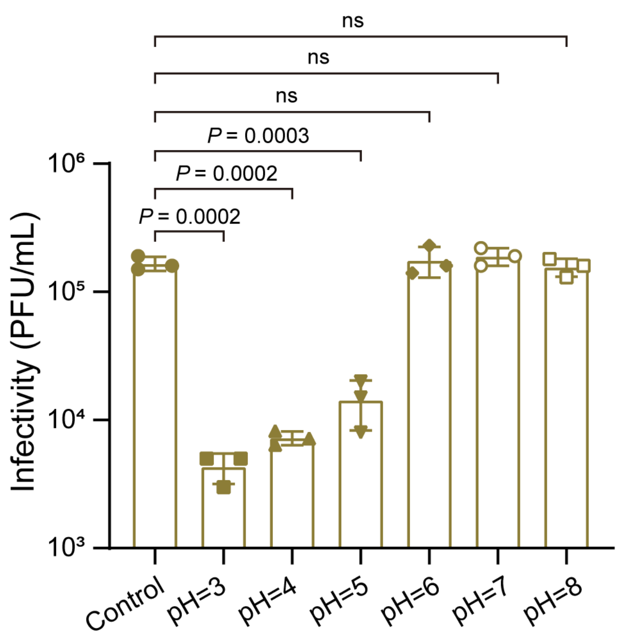

Supplementary Figure 16. Effects of MNPs@Ab on the infectivity of viruses at different pH values. MNPs@Ab (200 μg/mL) and H_2O_2 (1 mM) were incubated with EV71 at different pH values for 24 hours, and then the infectivity of EV71 was assessed by plaque forming assays. The data are presented as the mean ± s.d. (n = 3). Statistical significance was calculated via two-tailed Student's t test. $P < 0.05$ was considered significant. Ns, not significant.

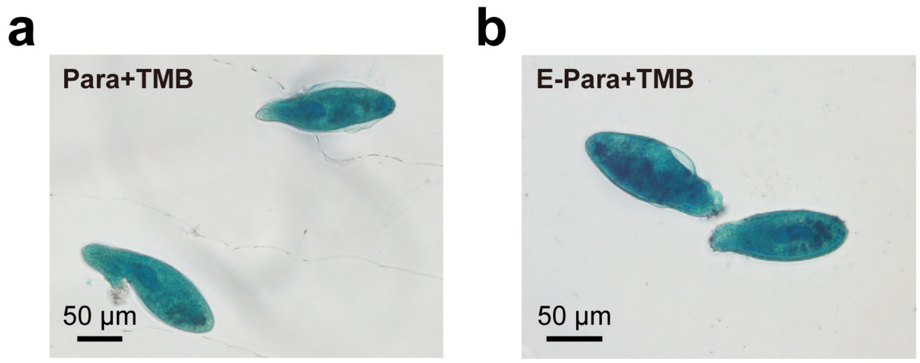

Supplementary Figure 17. Color reaction of Para/E-Para after addition of TMB.

Para (**a**) and E-Para (**b**) turned blue after colorless TMB was added.

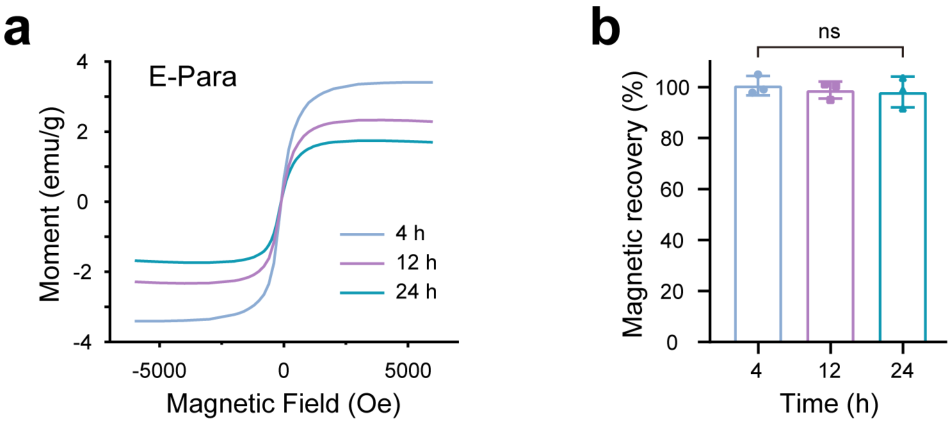

Supplementary Figure 18. Time dependence of the magnetism and magnetic recovery rate of E-Para. a Time-dependent magnetic hysteresis loop of E-Para. **b** Magnetic recoveries of E-Para for different periods. The data are presented as the mean ± s.d. (n = 3). Statistical significance was calculated via two-tailed Student's t test. $P < 0.05$ was considered significant. Ns, not significant.

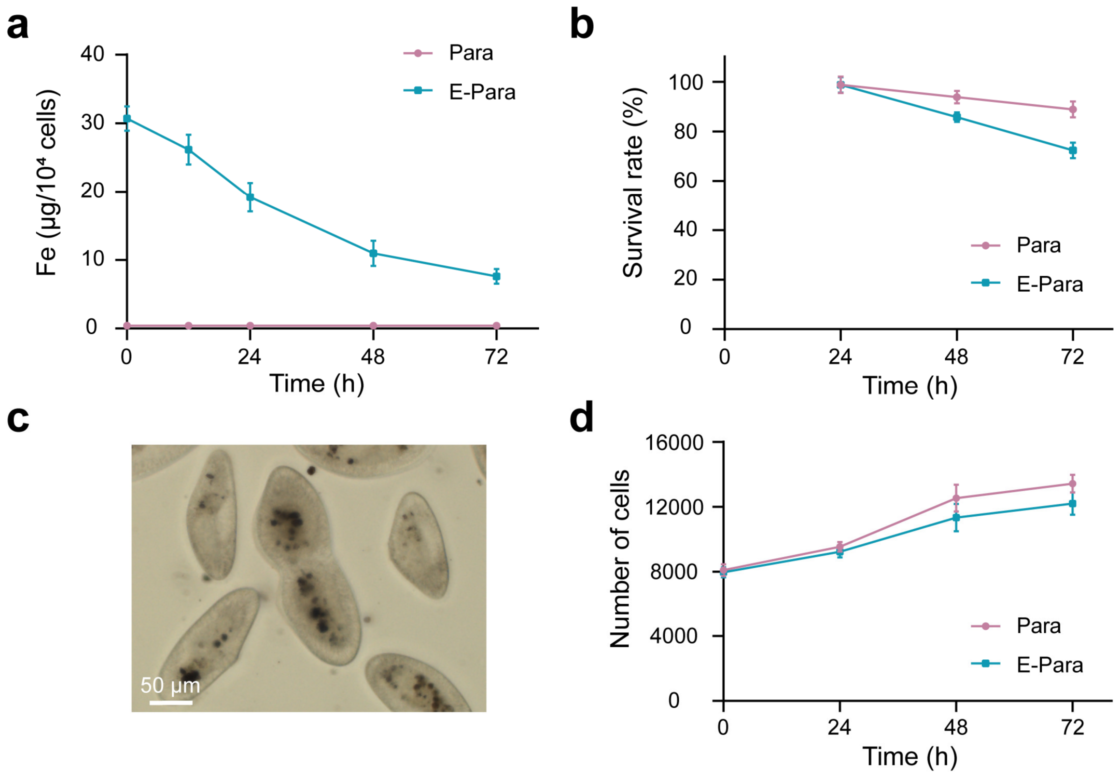

Supplementary Figure 19. The effect of MNPs@Ab on cytotoxicity and proliferation of E-Para. a Time dependence of Fe content in E-Para. The Fe inside E-Para after 12, 24, 48 and 72 hours was measured by ICP-MS. The data are presented as the mean ± s.d. (n = 3). **b** Survival rate of E-Para at different time. The survival rate was measured after culturing in KDS buffer at 25 °C for 24, 48 and 72 hours. In this experiment, all the virus capture assay was conducted in starvation condition, that is incubating in culture medium without food. The survival rate showed only 89% of Para and 72% of E-Para remained active after incubation for 72 h. The data are presented as the mean ± s.d. (n = 3). **c** Distribution of MNPs@Ab during the reproduction of E-Para. In the process of transverse division of E-Para, MNPs@Ab in E-Para are divided into two daughter cells. **d** Cell number of Para and E-Para under growth condition. Para and E-Para were cultured in medium containing *E.coli* at 25 °C for 24, 48 and 72 hours and the cell numbers were counted. The data are presented as the mean ± s.d. (n = 3).

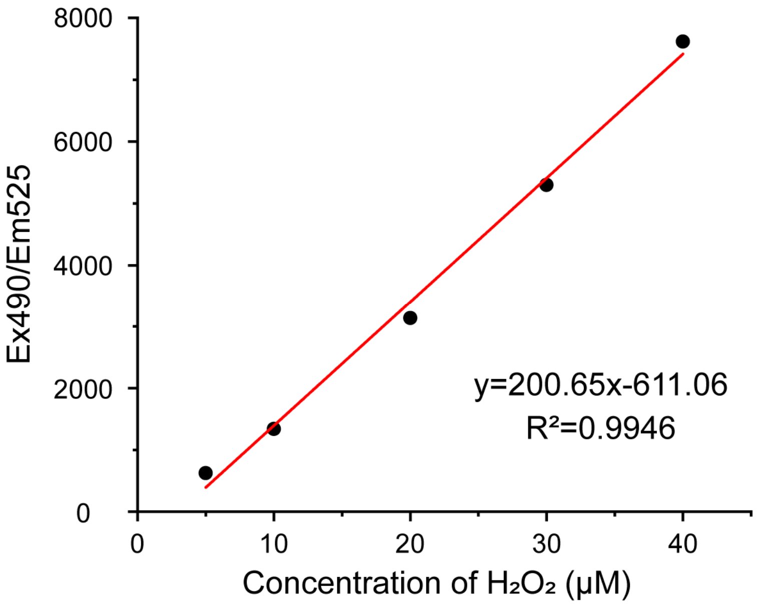

Supplementary Figure 20. Standard curve of H_2O_2. The standard curve for H_2O_2 was obtained by measuring the fluorescence intensity at 490 nm excitation and 525 nm emission of the mixtures of known concentrations of H_2O_2 and ROSGreen^{TM}.

Supplementary Tables

Supplementary Table 1. Conventional methods for eliminating viruses in water

Methods	Strength	Weakness
Membrane filtration (MF/UF/NF/RO)	Security, specific types of membranes are effective for virus removal	High processing pressures, unstable removal efficiency, health risk potential for humans
Chlorination	Easy to handle, economical	Disinfection by-product (DBP) formation, corrosive, residual toxicity
Ozonation	Short contact time, inactivation of viruses	High energy consumption, DBP
UV light radiation	No DBP formation, short contact time	Relatively high level of energy consumption, resistance of viruses to UV
Catalytic oxidation (Photocatalysis/Electrocatalysis)	Facile preparation, favorable catalytic Performance, low operation cost	Hydroxyl radical reduction caused by natural organic matter (NOM) affects disinfection efficiency

Supplementary Table 2. Primers used for the RT–qPCR analyses.

Primer	Sequence
EV71-F	GGCCATTTATGTGGGTAACTTTAGA
EV71-R	CGGGCAATCGTGTCACAAC
H1N1-F	GACCRATCCTGTCACCTCTGAC
H1N1-R	GGGCATTYTGGACAAAKCGTCTACG
Ad5-F	CGCCACCGAGACGTACTTCA
Ad5-R	TGTGGTCACGTCGTGCGTAG

Supplementary References

1. Xu, Y.-P. et al. Rational design of a replication-competent and inheritable magnetic viruses for targeting biomedical applications. *Small* **16**, 2002435 (2020).
2. Zhang, Y. et al. Conformation-stabilized amorphous nanocoating for rational design of long-term thermostable viral vaccines. *ACS Appl. Mater. Interfaces* **14**, 39873-39884 (2022).
3. Perez-Benito, J.F.J.J.o.P.C.A. Iron(III)−Hydrogen peroxide reaction: Kinetic evidence of a hydroxyl-mediated chain mechanism. *J. Phys. Chem. A* **108**, 4853-4858 (2004).
4. Zhu, Y. et al. Strategies for enhancing the heterogeneous Fenton catalytic reactivity: A review. *Appl. Catal. B-Environ.* **255**, 117739 (2019).
5. Qian, X., Zhang, J., Gu, Z. & Chen, Y. Nanocatalysts-augmented Fenton chemical reaction for nanocatalytic tumor therapy. *Biomaterials* **211**, 1-13 (2019).
