# Supplementary material for: Material-engineered bioartificial microorganisms enabling efficient scavenging of waterborne viruses
Source: Nat Commun. 2023 Aug 3;14:4658. doi: 10.1038/s41467-023-40397-5 (PMC10400550; doi:10.1038/s41467-023-40397-5)
Supplement: Supplementary file 2 — Reporting Summary [file 41467_2023_40397_MOESM2_ESM.pdf]

nature portfolio

Corresponding author(s): Xiaoyu Wang and Ruikang Tang

Last updated by author(s): Jul 26, 2023

Reporting Summary

Nature Portfolio wishes to improve the reproducibility of the work that we publish. This form provides structure for consistency and transparency in reporting. For further information on Nature Portfolio policies, see our Editorial Policies and the Editorial Policy Checklist.

Statistics

For all statistical analyses, confirm that the following items are present in the figure legend, table legend, main text, or Methods section.

n/a	Confirmed	
☐	☒	The exact sample size (*n*) for each experimental group/condition, given as a discrete number and unit of measurement
☐	☒	A statement on whether measurements were taken from distinct samples or whether the same sample was measured repeatedly
☐	☒	The statistical test(s) used AND whether they are one- or two-sided<br>*Only common tests should be described solely by name; describe more complex techniques in the Methods section.*
☒	☐	A description of all covariates tested
☒	☐	A description of any assumptions or corrections, such as tests of normality and adjustment for multiple comparisons
☐	☒	A full description of the statistical parameters including central tendency (e.g. means) or other basic estimates (e.g. regression coefficient) AND variation (e.g. standard deviation) or associated estimates of uncertainty (e.g. confidence intervals)
☐	☒	For null hypothesis testing, the test statistic (e.g. *F*, *t*, *r*) with confidence intervals, effect sizes, degrees of freedom and *P* value noted<br>*Give P values as exact values whenever suitable.*
☒	☐	For Bayesian analysis, information on the choice of priors and Markov chain Monte Carlo settings
☒	☐	For hierarchical and complex designs, identification of the appropriate level for tests and full reporting of outcomes
☒	☐	Estimates of effect sizes (e.g. Cohen's *d*, Pearson's *r*), indicating how they were calculated

Our web collection on statistics for biologists contains articles on many of the points above.

Software and code

Policy information about availability of computer code

Data collection	CLSM data was collected with FV1000-ASW (Version 4.1, Olympus).<br>PCR data was collected with Applied Biosystems 7500 Fast Real-Time PCR System Software (Version 7500 v.2.0.6, ThermoFisher).<br>Hydroxyl radical data was collected with Bruker Biospin WinEPR Software (Version 4.40.11.65, Bruker).<br>Absorbance data was collected with Gen5 (Version 3.08, Biotek).
Data analysis	All statistical analyses were performed using Graphpad Prism (Version 8) , OriginPro (Version 2018), ImageJ (Version 1.51j8) and Imaris (Version 9.6).

For manuscripts utilizing custom algorithms or software that are central to the research but not yet described in published literature, software must be made available to editors and reviewers. We strongly encourage code deposition in a community repository (e.g. GitHub). See the Nature Portfolio guidelines for submitting code & software for further information.

Data

Policy information about availability of data

All manuscripts must include a data availability statement. This statement should provide the following information, where applicable:

- Accession codes, unique identifiers, or web links for publicly available datasets
- A description of any restrictions on data availability
- For clinical datasets or third party data, please ensure that the statement adheres to our policy

The source data generated in this study are provided in the Source Data file. The full image dataset is available from the corresponding authors upon request.

Research involving human participants, their data, or biological material

Policy information about studies with human participants or human data. See also policy information about sex, gender (identity/presentation), and sexual orientation and race, ethnicity and racism.

Reporting on sex and gender	This information has not been collected.
Reporting on race, ethnicity, or other socially relevant groupings	This information has not been collected.
Population characteristics	This information has not been collected.
Recruitment	This information has not been collected.
Ethics oversight	This information has not been collected.

Note that full information on the approval of the study protocol must also be provided in the manuscript.

Field-specific reporting

Please select the one below that is the best fit for your research. If you are not sure, read the appropriate sections before making your selection.

[x] Life sciences [ ] Behavioural & social sciences [ ] Ecological, evolutionary & environmental sciences

For a reference copy of the document with all sections, see nature.com/documents/nr-reporting-summary-flat.pdf

Life sciences study design

All studies must disclose on these points even when the disclosure is negative.

Sample size	No sample-size calculations were performed. Sample sizes were chosen according to standard practice in the field.
Data exclusions	No data were excluded from the analyses.
Replication	All the experiments were conducted at least three times and could be reliably reproduced.
Randomization	All the organisms used in our experiments are grouped randomly.
Blinding	The investigators were blinded to group allocation during data collection and analysis.

Reporting for specific materials, systems and methods

We require information from authors about some types of materials, experimental systems and methods used in many studies. Here, indicate whether each material, system or method listed is relevant to your study. If you are not sure if a list item applies to your research, read the appropriate section before selecting a response.

Materials & experimental systems

n/a	Involved in the study	
☐	☒	Antibodies
☐	☒	Eukaryotic cell lines
☒	☐	Palaeontology and archaeology
☒	☐	Animals and other organisms
☒	☐	Clinical data
☒	☐	Dual use research of concern
☒	☐	Plants

Methods

n/a	Involved in the study	
☒	☐	ChIP-seq
☒	☐	Flow cytometry
☒	☐	MRI-based neuroimaging

Antibodies

Antibodies used	All the antibodies were diluted and used following the supplier's protocol.<br>For preparation of MNPs@Ab, Anti-Mouse Enterovirus 71 Monoclonal Antibody, Merck, cat. no. MAB979 was used.<br>For indirect immunofluorescence imaging, Anti-Mouse Enterovirus 71 Monoclonal Antibody, Merck, cat. no. MAB979 and Goat Anti-Mouse IgG (H+L) Highly Cross-Adsorbed Secondary Antibody conjugated with Alexa Fluor Plus 555, ThermoFisher, cat. no. A32727 was used.
Validation	Monoclonal Antibody: This Anti-Enterovirus 71 Antibody, cross-reacts with Coxsackie A16, clone 422-8D-4C-4D is validated for use in IF for the detection of Enterovirus 71.<br>Secondary Antibody: This Antibody was verified by Relative expression to ensure that the antibody binds to the antigen stated.

Eukaryotic cell lines

Policy information about cell lines and Sex and Gender in Research

Cell line source(s)	The rhabdomyosarcoma (RD) cells were purchased from Meisen Chinese Tissue Culture Collections (Zhejiang, China).<br>Experimental Paramecium (Paramecium caudatum), originally isolated from a lake in Zhejiang University campus (Hangzhou, Zhejiang, China), was established a monoclonal and cultured with a bacterial culture (Escherichia coli) used as food source in the laboratory.
Authentication	The RD cells lines were authenticated by Meisen Chinese Tissue Culture Collections (Zhejiang, China).<br>STR profiling:<br>Amelogenin: X CSF1PO:10,13 D13S317:11,12 D16S539:11,12 D5S818:12 D7S820:8,10 TH01:8,9.3 TPOX:8,9 vWA:15,17
Mycoplasma contamination	RD cell was tested for mycoplasma contamination. No mycoplasma contamination was found.
Commonly misidentified lines (See ICLAC register)	No commonly misidentified cell lines were used.
