# Supplementary material for: Material-engineered bioartificial microorganisms enabling efficient scavenging of waterborne viruses
Source: Nat Commun. 2023 Aug 3;14:4658. doi: 10.1038/s41467-023-40397-5 (PMC10400550; doi:10.1038/s41467-023-40397-5)
Supplement: Supplementary file 3 — Description of Additional Supplementary Files [file 41467_2023_40397_MOESM3_ESM.pdf]

Title: Supplementary Movie 1
Description: Movement of Para.

Title: Supplementary Movie 2
Description: Movement of E-Para.

Title: Supplementary Movie 3
Description: Magnetotaxis of E-Para.
